# Large-area transfer of two-dimensional materials free of cracks, contamination and wrinkles via controllable conformal contact

**DOI:** 10.1038/s41467-022-31887-z

**Published:** 2022-07-29

**Authors:** Yixuan Zhao, Yuqing Song, Zhaoning Hu, Wendong Wang, Zhenghua Chang, Yan Zhang, Qi Lu, Haotian Wu, Junhao Liao, Wentao Zou, Xin Gao, Kaicheng Jia, La Zhuo, Jingyi Hu, Qin Xie, Rui Zhang, Xiaorui Wang, Luzhao Sun, Fangfang Li, Liming Zheng, Ming Wang, Jiawei Yang, Boyang Mao, Tiantian Fang, Fuyi Wang, Haotian Zhong, Wenlin Liu, Rui Yan, Jianbo Yin, Yanfeng Zhang, Yujie Wei, Hailin Peng, Li Lin, Zhongfan Liu

**Affiliations:** 1 Center for Nanochemistry, Beijing Science and Engineering Center for Nanocarbons, Beijing National Laboratory for Molecular Science, College of Chemistry and Molecular Engineering, Peking University, Beijing, 100871 P. R. China; 2 Beijing Graphene Institute, Beijing, 100095 P. R. China; 3 Department of Physics and Astronomy, University of Manchester, Manchester, M13 9PL UK; 4 LNM, Institute of Mechanics Chinese Academy of Sciences, Beijing, P. R. China; 5 School of Engineering Sciences, University of Chinese Academy of Sciences, Beijing, 100049 P. R. China; 6 State Key Laboratory of Heavy Oil Processing, College of Science, China University of Petroleum, Beijing, 102249 P. R. China; 7 Academy for Advanced Interdisciplinary Studies, Peking University, Beijing, 100871 P. R. China; 8 CAS Key Laboratory of Nanosystem and Hierarchical Fabrication, CAS Center for Excellence in Nanoscience, National Center for Nanoscience and Technology, Chinese Academy of Sciences, Beijing, 100190 P. R. China; 9 Key Laboratory of Opto-Electronics Technology Ministry of Education College of Electronic Science and Technology Faculty of Information Technology, Beijing University of Technology, Beijing, 100190 P. R. China; 10 Beijing National Laboratory for Molecular Sciences, National Centre for Mass Spectrometry in Beijing, CAS Key Laboratory of Analytical Chemistry for Living Biosystems, Institute of Chemistry, Chinese Academy of Sciences, Beijing, 100190 P. R. China; 11 School of Materials Science and Engineering, Peking University, Beijing, 100871 P. R. China

**Keywords:** Electronic properties and devices, Graphene, Electronic properties and devices

## Abstract

The availability of graphene and other two-dimensional (2D) materials on a wide range of substrates forms the basis for large-area applications, such as graphene integration with silicon-based technologies, which requires graphene on silicon with outperforming carrier mobilities. However, 2D materials were only produced on limited archetypal substrates by chemical vapor deposition approaches. Reliable after-growth transfer techniques, that do not produce cracks, contamination, and wrinkles, are critical for layering 2D materials onto arbitrary substrates. Here we show that, by incorporating oxhydryl groups-containing volatile molecules, the supporting films can be deformed under heat to achieve a controllable conformal contact, enabling the large-area transfer of 2D films without cracks, contamination, and wrinkles. The resulting conformity with enhanced adhesion facilitates the direct delamination of supporting films from graphene, providing ultraclean surfaces and carrier mobilities up to 1,420,000 cm^2^ V^−1^ s^−1^ at 4 K.

## Abstract (web summary)

Reliable transfer techniques are critical for the integration of 2D materials with arbitrary substrates. Here, the authors describe a method to transfer 4-inch and A4-sized defect-free graphene films onto rigid and flexible substrates with controllable conformal contact, leading to improved electrical properties and uniformity.

## Introduction

Many efforts have resulted in the breakthrough in the chemical vapor deposition (CVD) fabrication of large-area graphene^1,2^ and other 2D matereials^3^ on archetypal growth substrates. However, a critical challenge has emerged in the lack of reliable after-growth transfer techniques for layering 2D membranes onto arbitrary substrates for large-scale technological applications^4–6^. Currently, the most significant issues associated with the 2D materials transfer arise from cracks, wrinkles, and interfacial contamination^7–9^ generated during the transfer that compromises the homogeneity and electronic performance, such as carrier mobility. Additionally, such difficulties grow exponentially with increasing film size^7^.

The flexibility and thinness of graphene make it susceptible to mechanical deformation and damage in conventional CVD graphene film transfer processes^10^. The key to achieving crack-free transfer is the continuous mechanical support for graphene during transfer. Such support can be supplied by introducing supporting films and the conformal contact between graphene and the target substrate^11,12^. In this regard, various supporting foils have already been introduced^8,9,13^. However, after the removal of supporting foils, non-conformal adherence of graphene onto the target substrate would make the graphene free-standing in some regions; such free-standing graphene would be torn by the interfacial forces in the subsequent removal of the transfer medium. As a consequence, a universal approach to realizing conformity between substrates and graphene is critically needed to achieve crack-free transfer.

In addition, the removal of supporting polymer films, such as poly (methyl methacrylate) (PMMA), is usually insufficient and requires aggressive chemical treatments^14^. Therefore, the removal procedure generates organic waste solvents and leaves unavoidable contamination on surfaces as well as metals and etchant residues^15^ in etching-based delamination, which degrades graphene quality^9,14^. Polymer-free techniques are efficient for avoiding contamination, which, however, fails in large-area transfer^16^. Hence, methods for contamination-free transfer that do not require the use of organic solvents are in high demand for large-area transfer.

Herein, by adding oxhydryl groups-containing volatile molecules (OVM) (cedrol, alpha-terpineol, linalool, and borneol) or low-glass-transition-temperature (*T*_g_) polymers (polypropylene carbonate, PPC) into PMMA, we can achieve the conformal contact between graphene and destination substrates, and the controllable conformal contact enables us to achieve the transfer of 4-inch graphene single-crystal wafers on Cu wafers^2,17^ and A4-sized graphene films on Cu foils^1,13^ (two research hotspots of CVD graphene^18^) onto rigid SiO_2_/Si and soft polyethylene terephthalate (PET) substrates, respectively, free of cracks, wrinkles, and contamination, which contribute to the improved electrical performance. Furthermore, the conformal contact improves graphene-substrate adhesion and allows for direct delamination of supporting films without producing contamination.

## Results

### Structural design of supporting films

If the contact with graphene is conformal, the substrate would support the graphene films by undertaking the interfacial forces, thereby avoiding the formation of cracks (Supplementary Fig. 1a, b). However, the rough topography of graphene films, which inherits from the corrugated structure of growth substrates after the delamination (Supplementary Fig. 1c, d) as well as the rough surface of substrates (Supplementary Fig. 1e, f) altogether make the formation of conformal contact difficult^11^. Therefore, the key to the successful transfer in achieving the conformal contact between graphene and substrates by the structural design of supporting films. Despite several references^11,12,16,19,20^ that mentioned the role of conformal contact in the crack-free transfer, surface contamination, cracks and wrinkles still existed in as-transferred graphene films with non-comparable carrier mobility to exfoliated counterparts, which indicates that the fine conformal contact over large area remains unachievable.

**Fig. 1:**
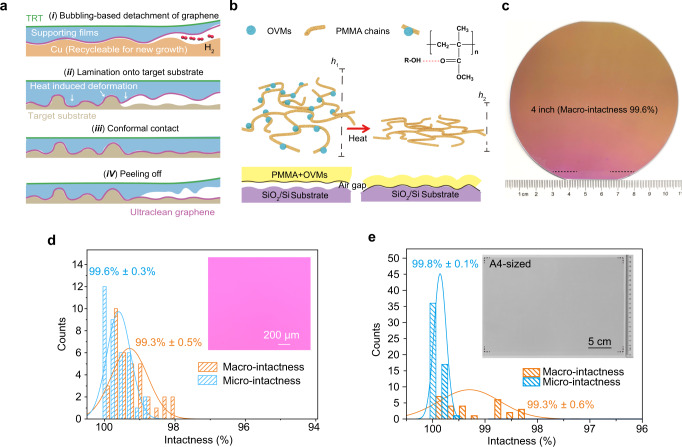
The crack-free transfer of large-area graphene films onto SiO_2_/Si wafers and polyethylene terephthalate (PET) substrates. **a** Illustration of techniques for transferring graphene onto destination substrates free of cracks and contamination. Before the bubbling-based separation, the lamination of the rigid thermal release tape (TRT) onto the supporting films is essential for large-scale operability. **b** Mechanism illustration of the heat-induced deformation and resulted conformal contact. Heat treatment would enable the evaporation of oxhydryl groups-containing volatile molecules (OVMs) and the height change (from h_1_ to h_2_) of the supporting films. **c** Photograph of 4-inch graphene single-crystal transferred onto SiO_2_/Si wafers. The region below the black dash line is not covered by graphene, because this region was not coated by supporting films for being connected with electrodes during bubbling delamination. **d**, **e** Statistics of Macro-intactness (orange) and Micro-intactness (blue) of as-transferred graphene on 4-inch SiO_2_/Si (**d**) and A4-sized PET (**e**). **d** Optical microscopy (OM) image of graphene on SiO_2_/Si substrates at 5× magnification. **e** A4-sized scanned image of graphene on PET. Note that graphene in **c**, **d** was transferred by cedrol (10 wt%)/PMMA.

By adding OVMs or PPC into PMMA, we can trigger the deformation of supporting films under heat and achieve the conformal contact between graphene and destination substrates with different surface contours. The controllable conformal contact improves graphene-substrate adhesion and allows for direct delamination of supporting films without producing contamination and cracks (Fig. 1a).

Owing to the roughness difference, the transfer onto SiO_2_/Si and PET substrates was conducted by using OVMs- and PPC- modified supporting films, respectively. In the OVMs-modified supporting films, OVMs were firstly embedded into the PMMA chains by forming the strong hydrogen bond between the oxhydryl groups in OVMs and the carbonyl oxygen in the PMMA chains^21^, and steric hindrance of OVMs would increase inter-chain spacing. By heating, the embedded volatile molecules can be evaporated, causing the PMMA chains to restack, and therefore the polymer deformation pushes graphene to conformally contact the SiO_2_/Si substrates (Fig. 1b). Note that, the formation of hydrogen bond between small molecules and PMMA has already been used to modify the glass transition temperature^22^ and fragility of PMMA^23^.

The layer-by-layer blade coating of PPC and PMMA forms a film wherein the PMMA and PPC chains are not completely blended together^24^. Upon heating over the *T*_g_ of PPC, the adequate blending of PPC and PMMA occurs with a polymer deformation, and the PPC/PMMA in a viscous state would smooth out the rough surface of graphene inherited from the rough growth substrates. Such polymer blending would also induce an obvious height reduction of the entire polymer films by PPC chains restacking and pushing the graphene surface to contact the PET substrate conformally (Supplementary Fig. 2a). However, thermal release tape (TRT) was used to assist the large-area transfer (see Methods section), and the presence of the rigid TRT that contact with the PPC/PMMA films would impede the conformal contact due to the adhesion between TRT films and polymer films. Therefore, by heating the TRT, the release of the polymer and graphene onto the substrate would contribute to the final conformal contact between graphene and substrate. After the thermal release of the TRT, we used silicone tape to successfully peel off the PPC/PMMA films from the graphene surface to obtain a clean surface. The heat-induced deformation of modified supporting films was confirmed by the observed height change of films. After heating, the supporting films exhibited a height decrease of ~30 nm and ~27 nm (~20% of the total height) for OVMs-modified supporting films and PPC/PMMA supporting films, respectively, in white light interference images (Supplementary Fig. 2b–e). In contrast, a very small height reduction was observed in PMMA-only films upon heating (Supplementary Fig. 2f, g).

### The crack-free transfer of graphene

The fine conformity ensures the crack-free transfer. Transfer-induced cracks can be roughly categorized into centimeter- and micrometer-sized cracks, which are caused by macroscopic unsuccessful lamination and local non-conformal contact of graphene with substrates, respectively (Supplementary Fig. 3a–f). Photographs with 12-megapixel resolution are sufficient for characterizing centimeter-scale cracks of graphene on SiO_2_/Si substrates, based on the contrast difference (Fig. 1c and Supplementary Fig. 3h). We instead propose using a commercial scanner (24-megapixel resolution) to enhance the contrast difference to visualize graphene on transparent PET substrates (Fig. 1e, inset and Supplementary Fig. 4). Optical microscopy (OM) images were taken at ×5 and ×50 magnifications to comprehensively characterize the micrometer-sized cracks (Fig. 1d, inset and Supplementary Fig. 3i). The high sampling representativeness of the characterizing approach was confirmed by the very narrow distribution of obtained intactness (Supplementary Fig. 5). The final intactness of the transferred graphene (the product of the two intactness) is ~99% on both SiO_2_/Si and PET substrates (Fig. 1d, e).

To improve the productive capacity, we developed customized bubbling-delamination equipment with controllable delamination rate and force (Supplementary Fig. 6). The bubbling-based delamination would enable the recycling of the metal substrates for regrowth and avoid producing the waste of etching solution^25^. The entire time consumed in the transfer is also clearly reduced compared to conventional routes^15,26^ (Supplementary Tables 1 and 2), demonstrating the compatibility of our transfer route with industrial batch processing.

### The contamination-free transfer of graphene

**Fig. 2:**
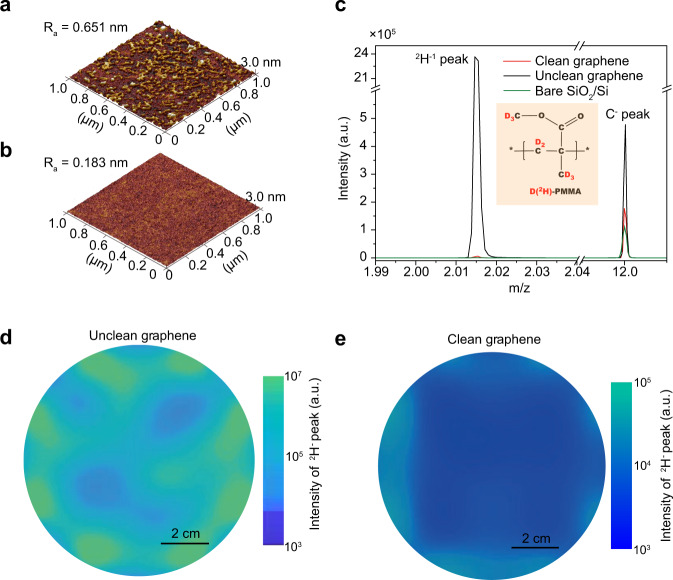
The contamination-free transfer of large-area graphene films. **a**, **b** atomic force microscopy (AFM) images of as-transferred graphene on SiO_2_/Si substrates by conventional PMMA-based techniques (**a**) and OVM-modified PMMA (**b**). R_a_: average roughness. **c** time-of-flight secondary ion mass spectroscopy (ToF-SIMS) spectra of as-transferred graphene on SiO_2_/Si substrates by conventional poly (methyl methacrylate) (PMMA)-based techniques (blue line), OVM-modified PMMA (red line), and bare substrate for reference (green line). Inset: structural formula of ^2^H^-^PMMA. **d**, **e** 4-inch mapping of ^2^H^−^ peak intensities of as-transferred graphene on SiO_2_/Si substrates by conventional PMMA-based techniques (**d**) and OVM-modified PMMA (**e**). Note that graphene in (**b**) was transferred by cedrol (10 wt%)/PMMA and in (**c**, **e**) was transferred by alpha-terpineol (10 wt%)/PMMA.

In the conventional transfer process, the difficulty in completely removing supporting films, inevitably causes surface contamination^8,9,14^ (Fig. 2a). While recently, there has been the development of new supporting films with high dissolvability in acetone, the basic idea behind eliminating residues should still be based on directly peeling supporting films off from graphene, such peeling process requires stronger adhesion between graphene and substrates than that between graphene and polymer, which was achieved by forming conformal contact of graphene with underlying substrates. Relying on the modified supporting film, we successfully improved conformity and adhesion. Therefore, we obtained clean graphene surfaces on SiO_2_/Si substrates by mechanically peeling off the supporting films, as evidenced by atomic force microscopy (AFM) images (Fig. 2b and Supplementary Fig. 7a–h). The corresponding height distribution of resulted clean surface is similar to that of bare substrates (Supplementary Fig. 8a)^27^. In contrast, owing to the presence of few-layer contamination, a broader peak in height distribution was observed for the unclean surface (conventional PMMA-based method), along with a side peak arising from the higher residue particles (Supplementary Fig. 8b). In addition, by using a white light interferometer, a comprehensive investigation of the roughness over the entire 4-inch sized wafer was conducted, and the as-obtained roughness is 0.25 ± 0.03 nm, which is similar with bare substrates (Supplementary Fig. 7i).

The contamination concentration and spatial distribution over wafer-scale were evaluated by using deuterium-labeled PMMA, which can be identified through time-of-flight secondary ion mass spectroscopy (ToF-SIMS) (Fig. 2c)^28^. Wafer-scale mapping of the deuterium peak intensity can reflect the spatial distribution of PMMA residues. Clearly, over the entire transferred single-crystal graphene wafer, the concentration of PMMA residues decreased by approximately four orders of magnitude when the supporting films were directly peeled off (Fig. 2d, e and Supplementary Fig. 8c). In addition, the as-transferred graphene exhibited no-residue-related C–O and O–C = O peak in X-ray photoelectron spectroscopy (XPS) results^9^, and exhibited similar peak with that of bare substrates, confirming the improved cleanness (Supplementary Fig. 8d–i). The conformal state also avoids the formation of new wrinkles; thus, we obtained 4-inch wrinkle-free graphene single crystals on SiO_2_/Si substrates relying on the suppression of wrinkle formation during both growth and transfer (Supplementary Fig. 8j–l).

### Conformity of transferred graphene with target substrates

**Fig. 3:**
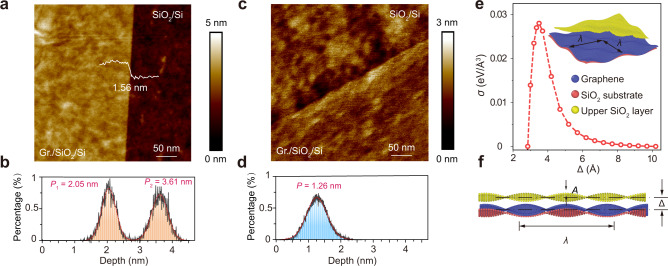
The conformal contact of graphene with destination substrates. **a**–**d** AFM image and height histogram of as-transferred graphene edges on SiO_2_/Si substrates by conventional PMMA-based techniques (**a**, **b**) and OVMs-modified supporting films (cedrol (10 wt%)/PMMA) (**c**, **d**). The distance between two peaks in the height histogram indicates the height of the as-transferred graphene edge is 1.56 nm by the conventional PMMA-based method. In contrast, the single peak in the height histogram of clean graphene edge reflects the smaller height difference across the edge and the fine conformity. **e**–**f** The adhesion in the collective interaction between atoms in graphene and the SiO_2_/Si substrates as a function of the distance between graphene and substrates (**e**) and the front view of the corresponding model structure (**f**). **e** the overlook view of the corresponding model structure. Δ is the average separation distance between the graphene and SiO_2_/Si layer. *A* and *λ* are the amplitude and wavelength of the surface corrugations, respectively.

Even if graphene is contacted with substrates in a dry environment, incomplete conformity can produce air gaps full of oxygen and water molecules, which is responsible for the widely observed *p*-doping transport behavior of graphene^26^. The presence of air gaps was confirmed by the large difference in height between graphene and the substrate in AFM images (Fig. 3a, b and Supplementary Fig. 9a, b, h). In contrast, when graphene conformally replicates the surface contours of the underlying substrate using OVMs-modified supporting films, such gaps are invisible with smaller height differences across the edge (Fig. 3c, d and Supplementary Fig. 9c, d). The observed fine conformity is similar to the case of exfoliated graphene (Supplementary Fig. 9e–g). In addition, the lamination of graphene onto destination substrates was also conducted in a dry environment; trapped water molecules could be further avoided^26^.

According to the stress-separation relationship in contact mechanics, the resulted conformity with the reduced separation distance between graphene and substrates would engineer the adhesion, enabling the mechanical delamination of supporting films. In the case of graphene on SiO_2_/Si substrates, graphene can be roughened by both thermal undulations and substrate interaction. The roughness of graphene (${z}_{C}$) can be approximated as follows:

1 $${z}_{C}=\sum\,{A}_{{mn}}\,{{\sin }}\frac{2\pi m}{{L}_{x}}x\,{{\sin }}\frac{2\pi n}{{L}_{y}}y$$

where *m* and *n* are both positive integers and ${A}_{{mn}}$is the amplitude of the corresponding displacement. ${L}_{x}$ and ${L}_{y}$ are the lengths of the graphene sheet. The roughness of SiO_2_/Si substrates can be depicted by a sinusoidal function:

2 $${z}_{{{{{{\rm{Si}}}}}}{{{{{{\rm{O}}}}}}}_{2}}=\Delta +{A}_{{{{{{\rm{Si}}}}}}{{{{{{\rm{O}}}}}}}_{2}}{{\sin }}\frac{2\pi }{\lambda }x\,{{\sin }}\frac{2\pi }{\lambda }y$$

where $\Delta$ is the average separation distance between the graphene and SiO_2_/Si layer. ${A}_{{{{{{\rm{Si}}}}}}{{{{{{\rm{O}}}}}}}_{2}}$ and $\lambda$ are the amplitude and wavelength of the surface corrugations, respectively. Hence, the separation distance between SiO_2_ and graphene at position (x, y) is ${\Delta }_{z}={z}_{{{{{{\rm{Si}}}}}}{{{{{{\rm{O}}}}}}}_{2}}-{z}_{{{{{{\rm{C}}}}}}}$. Statistically, adhesion stress ($\left\langle \sigma \left(\triangle \right)\right\rangle$, adhesion force per unit area) can be described by^29^:

3 $$\left\langle \sigma \left(\triangle \right)\right\rangle =-\int \rho ({\Delta }_{z})\frac{\partial E\left({\Delta }_{z}\right)}{\partial {\Delta }_{z}}d{\Delta }_{z}$$

where $\rho ({\Delta }_{z})$ represents the spatial distribution of ${\Delta }_{z}$ at the atomic scale, $-\frac{\partial E\left({\Delta }_{z}\right)}{\partial {\Delta }_{z}}$ is the corresponding van der Waals force. Therefore, the change of adhesion stress as a function of the separation distance would be similar to that of van der Waals force, in which after the peak at 3.2 Å, the stress would sharply reduce with increasing $\triangle$. Such relationship was also confirmed by a series of molecular dynamics (MD) simulations (Fig. 3e, f and Supplementary Fig. 9i). Therefore, the reduced separation distance in our case would contribute to a stronger adhesion.

### Properties of transferred graphene

**Fig. 4:**
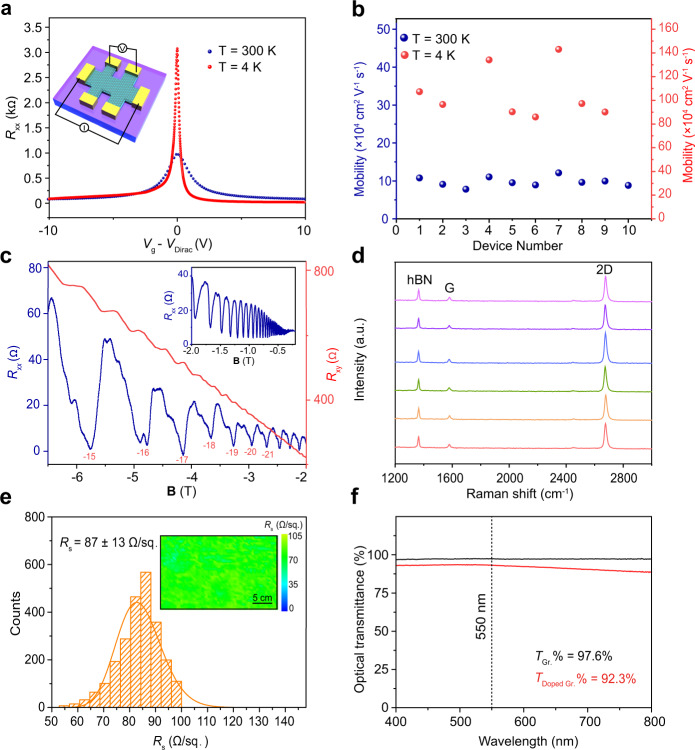
The properties of transferred graphene. **a** Typical transfer curves of as-transferred graphene after encapsulation by hBN and one-dimensional contacting at 300 K (navy blue) and at 4 K (red). Inset: Illustration of the measured Hall bar devices of encapsulated graphene. **b** The statistics of obtained carrier mobilities of the encapsulated graphene at 300 K (navy blue) and at 4 K (red). **c** *R*_xx_ (navy blue) and *R*_xy_ (red) as a function of magnetic field (**B**) at a fixed gate voltage (−50 V). Inset: *R*_xx_ as a function of **B** scanning from −0.2 to −2 T. *R*_xx_ is the longitude resistance, which can be obtained according to the equation: *R*_xx_ = *V*_xx_/*I*_ds_, and *R*_xy_ is the Hall resistance which can be obtained by *R*_xy_ = *V*_xy_/*I*_ds_. **d** Raman spectra of as-transferred graphene encapsulated by hBN. **e** Sheet resistance statistics of graphene on PET substrates doped by PEDOT:PSS. Inset: Corresponding A4-sized sheet resistance mapping of the doped graphene. **f** Ultraviolet-visible (UV-Vis) transmittance spectra of graphene films (black) and doped graphene films by PEDOT:PSS on PET substrates (red). Note that graphene in (**a**, **c**) was transferred by cedrol (10 wt%)/PMMA; devices No. 1, 2 were transferred by borneol (10 wt%)/PMMA; devices No. 3, 4, and 5 were transferred by alpha-terpineol (10 wt%)/PMMA; devices No. 6, 7, 8 were transferred by linalool (10 wt%)/PMMA and devices No.9, 10 were transferred by cedrol (10 wt%)/PMMA. Their corresponding Raman spectra were shown in (**d**). Note that the mass fraction of borneol in n-heptane should not be higher than 10% to avoid precipitating from the PMMA solution.

The crack-free transfer can be reflected by the noise-level D band intensity of the as-transferred graphene in the Raman results^29^ (Supplementary Fig. 10a). Moreover, the removal of contamination, wrinkles, and air gaps ensures a low full width at half maximum (FWHM) of the 2D band (~24 cm^−1^), which is an indicator of strain and doping level (Supplementary Fig. 10b)^30^. To evaluate its electronic quality, we encapsulated graphene by hexagonal boron nitride (hBN) to exclude substrate interference^31^, and the obtained carrier mobilities range from 70,000 to 120,000 cm^2^ V^−1^ s^−1^ at room temperature and from 800,000 to 1,420,000 cm^2^ V^−1^ s^−1^ at 4 K (Fig. 4a, b). These results are higher than previously reported values for CVD-graphene devices^32–34^ and among the best results of exfoliated and suspended graphene^35–37^. In addition, well-defined Shubnikov-de Haas oscillations of *R*_xx_ with full breaking of the fourfold degeneracy were observed at low temperatures (Fig. 4c)^38^. The enhanced carrier mobilities can also be reflected by low FWHM of 2D band (~17 cm^−1^) and high-intensity ratio of 2D band to G band (~8) in encapsulated graphene (Fig. 4d and Supplementary Fig. 10b, c). The determination of the field effect transistor carrier mobility values of graphene on SiO_2_/Si was conducted by fabricating Hall bar devices with 1.2 cm interval over the entire 4-inch wafer (inset, Supplementary Fig. 11b). It was found that the average value of 18 devices is 8800 cm^2^ V^-1^ s^−1^ at the room temperature (Supplementary Fig. 11a, b), which is relatively higher than the previous reports^11,12,16,19,20^.

The improved carrier mobility is caused by the reduced density of crack/contamination/wrinkle, suppressed water- and oxygen-related doping, and the releasing of the compressive strain by forming conformal contact between graphene and substrates (Supplementary Fig. 10d). Admittedly, the conformal contact would still introduce tensile strain in comparison with free-standing graphene^39^, which might be further optimized by interfacial design^40,41^.

The conformity of nanoscale films on flexible substrates is essential for reliable flexible electronics^42^. Additionally, conductivity and optical transmittance are two other performance parameters in this field^43^. The improved intactness and cleanness of graphene in our method ensure the reduced sheet resistance compared to the conventional PMMA-based transfer method (Supplementary Fig. 12a, b). In addition, after the transfer, the blade coating of poly(3,4-ethylenedioxythiophene) (PEDOT): polystyrene sulfonate (PSS) enabled the uniform reduction of the sheet resistance over a large area to 87 ± 13 Ω/sq. with an optical transmittance of 92.3% (Fig. 4e, f and Supplementary Fig. 12c).

### General transfer of nanoscale films

**Fig. 5:**
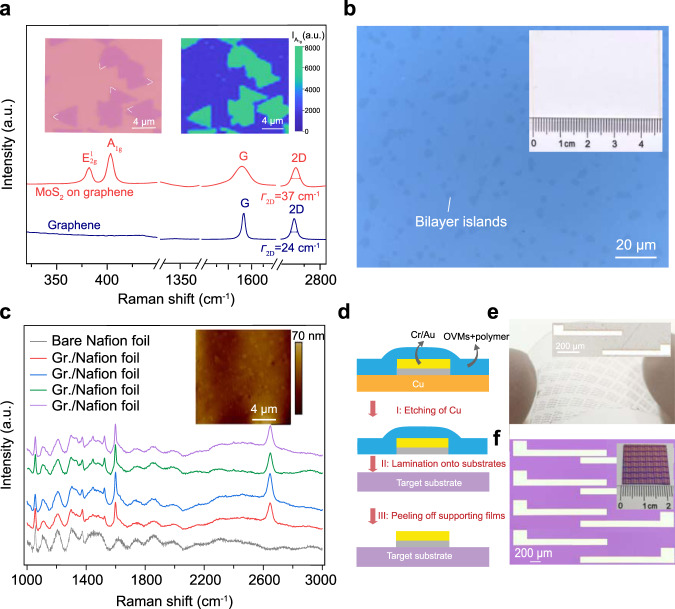
General transfer of nanoscale films. **a** Raman spectra of MoS_2_/graphene heterostructure (red) and bare graphene (navy blue) on SiO_2_/Si substrates. Inset: OM image of CVD-grown monolayer MoS_2_ islands transferred onto graphene and corresponding Raman mapping of A_1g_ band intensity. **b** OM image of graphene transferred onto Nafion foils. Bilayer islands would enable the visualization of monolayer graphene region. Inset: photography of the graphene transferred onto Nafion foils. **c** Raman spectra of graphene transferred onto Nafion foils and bare Nafion foils (gray). Inset: AFM image of transferred graphene on Nafion foils. **d** Illustration of the fabrication of the transferable Cr/Au electrodes and transfer of the electrodes onto other substrates using the OVMs-modified supporting films. **e** Photograph of transferred Cr/Au electrodes on PET substrate. Inset: OM image of transferred Cr/Au electrodes. **f** OM image of transferred Cr/Au electrodes on SiO_2_/Si substrates. Inset: photograph of transferred Cr/Au electrodes on SiO_2_/Si substrates. Note that graphene in (**a**) was transferred by cedrol (10 wt%)/PMMA; graphene in (**b**, **c**) was transferred by borneol (10 wt%)/PMMA; graphene in (**d**) was transferred by alpha-terpineol (10 wt%)/PMMA.

Reliable transfer techniques that ensure intact and clean interfaces are crucial for the fabrication of van der Waals heterostructures with new functions and unprecedented performance^44^. To demonstrate the capability of our transfer method in van der Waals integration, we fabricated a graphene/monolayer molybdenum disulfide (MoS_2_) vertical heterostructure via layer-by-layer transfer (Fig. 5a, inset). In the Raman spectra, a uniform distribution of the A1g (out-of-plane vibration) and E^1^_2g_ (in-plane vibration) intensities confirmed the successful transfer of MoS_2_ onto graphene^45,46^ (Fig. 5a, inset and Supplementary Fig. 13a). Additionally, no D band was observed over the imaging region, indicating that no new wrinkles or cracks were formed in graphene during MoS_2_ transfer (Supplementary Fig. 13b)^29^. A clean interface and fine conformity in the van der Waals heterostructure cause a strong electronic interaction between layers, such as photoinduced electron transfer^45–48^. In our case, uniform blueshift of the G band and redshift of the 2D band were observed, consistent with the results of the high-temperature epitaxially grown MoS_2_ on graphene (Supplementary Fig. 13c–e)^45^. This confirms the strong interlayer coupling in as-fabricated heterostructure. In addition, strong compressive strain incurred by the conformal contact as evidenced by the broadening of the graphene 2D band (Supplementary Fig. 13f)^30,48^.

A universal transfer technique requires its compatibility with various subject materials and target substrates, especially for the substrates that cannot survive in organic solvents, such as Nafion foils. The proposed organic solvent-free transfer enabled us to transfer graphene onto Nafion foils without damaging graphene and substrates, as confirmed by the OM images and the clear 2D and G bands in the Raman analysis (Fig. 5b, c). The graphene/Nafion foil structure is promising for future hydrogen isotope separation^49,50^. Furthermore, based on the artificial design of supporting films and the use of Cu as a sacrificial layer, we were able to transfer gold electrodes with a thickness of tens of nanometers onto various substrates without chemical reactions to release electrodes (Fig. 5d–f).

## Discussion

In summary, our work demonstrates a method for forming conformal contact, and interfacial adhesion engineering in 2D regime. Controllable conformity between graphene and target substrates was readily realized by the structural design of supporting films, and fine conformity allows us to remove the supporting films mechanically without producing new cracks or contamination over a large area. In addition, the as-transferred graphene exhibited improved carrier mobility comparable to exfoliated one. Because both organic solvent waste disposal and the etching of the growth substrates were avoided in our method, it can be easily modified for future industrial production.

## Methods

### Preparation of single-crystal Cu (111) wafer on sapphire

Single-crystal sapphire wafers (4-inch, c plane, 500 μm thickness and 6-inch, c plane, 1000 μm thickness) were utilized as the epitaxy substrates for the preparation of single-crystal Cu. Before the deposition, sapphire wafer was annealed in a pure oxygen atmosphere for 6 h at 1020 °C in atmosphere pressure to achieve an oxygen-terminated surface to reduce the density of in-plane twin boundaries in Cu films. Subsequently, a 500-nm-thick Cu film was deposited on sapphire wafer by radio frequency (RF) sputtering equipment (200 W RF power, and 0.5 nm/s deposition rate). Then, to prepare single-crystal Cu (111), Cu/sapphire wafer was annealed at 1020 °C for 2 h in atmosphere pressure with 1000 sccm Ar and 100 sccm H_2_.

### Growth of single-crystal graphene wafers

The Cu (111) wafer was first heated to 1020 °C in an atmosphere-pressure CVD system with 1000 sccm Ar and 100 sccm H_2_. Subsequently, 100 sccm CH_4_ (0.1% diluted in Ar) was introduced to initiate the graphene growth, and graphene wafer with full coverage could be obtained after 2 h. After graphene growth, the system was cooled down to room temperature under the same gas flow.

### Growth of graphene on Cu-Ni wafers

The Cu_90_Ni_10_ (111) wafer was first heated to 1020 °C in atmosphere-pressure CVD system with 1000 sccm Ar and 400 sccm H_2_. Subsequently, 2 sccm CH_4_ was introduced to initiate the graphene growth, and graphene wafer with full coverage could be obtained after 2 h growth. After the graphene growth, the system was cooled down to room temperature under the same gas flow.

### Growth of large-area graphene films on Cu foil

The large-area graphene films were grown using the low-pressure CVD system. The Cu foil (50 μm thick, Kunshan Luzhifa Electronic Technology Co., Ltd) was loaded into the tube furnace as the growth substrate. The sample was first heated to 1020 °C with 500 sccm Ar, followed by annealing with 500 sccm H_2_ for 30 mins. Then, the growth of graphene film was initiated by the introduction of 1 sccm CH_4_. After 1 hour growth, the system was cooled down to room temperature under the same flow.

### Transfer of graphene wafers onto SiO_2_/Si substrates

First, OVMs including cedrol (98% purity, Konoscience), alpha-terpineol (98% purity, Ark Pharm), linalool (95% purity, Leyan) and (-)-borneol (>97% purity, Alfa Aesar) were dissolved in n-heptane (Tianjin Concord Technology) (10 wt%), and the solutions were subsequently spin-coated on 4-inch graphene films grown on single-crystal Cu wafers. Thereafter, PMMA (950 K A4, Microchem Corp.) was spin-coated onto as-deposited OVMs layers to form a composite film by evaporation of the anisole (The thickness of PMMA is measured to be approximately 96.5 nm after the spin coating of PMMA on OVMs; The thickness of OVM is around 290 nm after spin-coating, and 215 nm after the drying. Note that the OVMs would be dissolved into the PMMA films that were spin-coated subsequently. Therefore, the final thickness would be less than 215 nm). Note that the anisole can also readily dissolve OVMs. Second, TRT (No.3198MS, Nitto Denko company. Note that other TRT tapes, including No.3195MS, No.319Y-4M, and No.3195 V are also applicable) is laminated onto the supporting films with a commercial laminator at room temperature (GMP A3 Laminator Machine 320, LSI) with a laminating rate of 2 cm per second. Subsequently, bubbling-based delamination of graphene from Cu is conducted by the customized bubbling-delamination equipment (see Supplementary Fig. 6). Note that the oxidation of the Cu substrates is not required in the bubbling-based delamination. After rinsing graphene with deionized water to remove residual electrolyte, the films would be dried in an oven at a temperature of 40 °C. Graphene was subsequently laminated on SiO_2_/Si substrates by the commercial laminator at the temperature of 100 °C. Heat treatment of graphene films at a temperature of 120 °C was conducted in oven to initiate the deformation of supporting films and the conformal contact. Note the heat treatment temperature can be reduced with longer heating time. Subsequently, supporting films/TRT was mechanically peeled off from graphene to obtain a clean graphene surface. The time consumed in the transfer is listed in Supplementary Table 2.

### Transfer of graphene films onto PET substrates

First, the A4-sized graphene film on Cu foil was blade coated with PPC (Mw = 30w, 0.05 g/ml, Aladdin Corp.) and PMMA (950 K A4, Microchem Corp.) layer-by-layer as the supporting films (15 mm/s) by a commercial blade coater (1811 BEVS). Subsequently, TRT was laminated onto the supporting films by a commercial laminator (GMP A3 Laminator Machine 320, LSI) at room temperature with a laminating rate of 2 cm per second. Subsequently, bubbling-based delamination of graphene from Cu was conducted by the customized bubbling-delamination equipment (see Supplementary Fig. 6). After rinsing graphene with deionized water to remove residual electrolyte and drying films in the oven at the temperature of 40 °C, the graphene film was laminated onto PET substrates by the commercial laminator at room temperature with the laminating rate of 2 cm per second.

Heating the films at temperature of 140 °C within one minute would result in the release of TRT. After the lamination of silicone tape onto the supporting films, we can peel off the silicone tape along with the supporting films and leave the clean graphene on PET. The time consumed in the transfer is listed in Supplementary Table 1.

### Coating of PEDOT:PSS onto graphene/PET

PEDOT:PSS (PH1000, 1-1.3 wt%, Heraeus) was sonicated (28 kHz) for 30 mins and filtered through a commercial filter to avoid large particles. Subsequently, 4.5 wt% of Dimethyl sulfoxide (DMSO) (99.9%, Sigma-Aldrich) and Zonyl FS-300 (0.5 wt%, Fluka) were added into PEDOT:PSS solution, and mixed overnight at room temperature. The DMSO was used to improve the electrical conductivity of PEDOT:PSS solutions and the Zonyl was used as a surfactant in mixed solution. The mixed solution was spin-coated onto graphene on PET at 1000 rpm for 60 s, followed by heating at 80 °C for 5 mins for the membrane formation.

### Growth and transfer of Monolayer MoS_2_ on Au Foil

Commercially available polycrystalline gold foil (99.99%, 30 μm thick) was cleaned by ultrasonication in hydrochloric acid (20 wt%) and acetone, respectively. Then the cleaned Au foil was annealed at 950 °C for 5 h. The annealed Au foil was faced downward to the MoO_3_ powder (~99.9%, ∼2 mg) in the heating center of the furnace (Lindberg/Blue M HTF55347c). The sulfur powder (~99.5%, 80 mg) was placed in a quartz boat at the upper stream, 30 cm away from the substrate. Monolayer MoS_2_ was grown at 750 °C for 8 min by ambient pressure CVD, and 80 sccm Ar was used as carrier gas. The same procedures (OVMs-modified supporting films) were used in the transfer of MoS_2_ onto pre-transferred graphene to fabricate the vertical heteostructure.

### Transfer of Cr/Au electrodes

The Cu wafer was used as the sacrificial layer for transferring the Cr/Au electrodes. Wafer-scale patterning on Cu wafers was conducted by laser direct writing (Heidelberg MLA-150) (photoresist, AR-P5350). Subsequently, the 5 nm Cr layer was deposited on Cu wafers, followed by the deposition of 50 nm Au using the e-beam evaporator (Angstrom Engineering nexdep). After the lifting off, coating of OVMs and PMMA was conducted to form supporting films. Subsequently, Cu wafer was etched away, leaving the Cr/Au electrodes on supporting films. After the rinsing and drying of the films, the films were laminated onto substrates such as PET and SiO_2_/Si substrates, followed by the direct peeling of supporting films off the substrates.

### PMMA-based transfer assisted by TRT

First, the PMMA solution (PMMA, 950 K A4, MicroChem Corp) was spin-coated onto one side of the graphene/Cu sample with spin-coating rate of 1000 rpm for 60 s and dried for 3 mins on a hot plate at the temperature of 80 °C. Oxygen plasma was used to etch graphene on the back side of the sample. Subsequently, we laminated the TRT onto PMMA film after the curing of PMMA. Then, electrochemical bubbling-based delamination of graphene from Cu was conducted, followed by rinsing and drying of the TRT/PMMA/graphene. Then we laminated graphene onto target substrates in a dry environment. After the lamination, heat treatment of the TRT would significantly reduce the adhesion energy between TRT and PMMA, which would leave the PMMA/graphene onto the target substrates. Finally, the PMMA was removed by soaking the sample in the acetone bath, and graphene was dried with compressed nitrogen gas.

### Transfer of graphene by conventional PMMA-based method

First, the PMMA solution (PMMA, 950 K A4, MicroChem Corp) was spin-coated onto one side of the graphene/Cu sample with a spin-coating rate of 1000 rpm for 60 s and dried for 3 mins on a hot plate at the temperature of 80 °C. Oxygen plasma was used to etch the graphene on the back side of sample. Subsequently, sodium persulfate (1 mol/L, Sigma-Aldrich) solution was used to etch Cu foil, and the PMMA/graphene film was floated on the surface of the solution. The PMMA/graphene film was washed with distilled water several times to remove the etchant residue. After the rinsing of the PMMA/graphene film with distilled water, the PMMA/graphene film was scooped out by a SiO_2_/Si substrate at room temperature and then was dried overnight to reduce the water trapped between graphene and substrates. Finally, the PMMA was removed by soaking the sample in the acetone bath and dried with compressed nitrogen gas.

### ToF-SIMS measurement

The ^2^H-PMMA is purchased from Polymer source company (production number #P100226-d5 PMMA) with *M*_n_ = 820,000 and *M*_w_ = 1,500,100. Annealing experiments were performed in the chamber of the ToF-SIMS spectrometer (ToF-SIMS V, ION-ToF GmbH, Munster, Germany) before characterization. The samples were analyzed at 25 °C after annealing at 100 °C for 1 h. ToF-SIMS spectra were acquired at the annealing temperature using a Bi_3_^+^ beam operating at 25 keV. The scanning area was 200 μm × 200 μm with an acquisition time of 40 s. Negative ion spectra were collected for each sample. The software used for peak analysis was SurfaceLab 6.0 from ION-ToF.

### Optical measurement

Optical microscopy images were obtained with a Nikon Olympus LV100ND. Raman spectra were obtained with LabRAM HR-800 with 532 nm laser. Optical transmittance spectra were collected by a Perkin-Elmer Lambda 950 UV-vis spectrophotometer. AFM characterization of graphene was carried out on Bruker dimension icon microscopy using the Scanasyst mode. The sheet resistance was measured using a four-probe system (CDE ResMap 178) based on the four-point probe method to eliminate contact resistance. Four metal probes were aligned in a line at intervals of 1 mm. White Light Interference images were conducted using a Nikon White Light Interferometry (BW-S501).

### Intactness characterization

Macro-intactness of graphene on SiO_2_/Si wafer was probed by taking the photographs of entire graphene wafer, while a commercial scanner was used to enhance the contrast difference to visualize graphene on transparent PET substrates for probing Macro-intactness. After taking the photos, the ratio of pixels was counted with different contrast to obtain the macro-intactness. OM images with 5× and 50× magnifications were taken to probe the micro-intactness: the twenty-five OM images with 5× magnifications were taken over transferred graphene (top, bottom, middle, right, and left) and we can obtain an average value. Subsequently, the OM images with 50× magnification were taken by randomly zooming in. After obtaining the values of macro-intactness and the micro-intactness using 5× and 50× magnification OM images, the three values were multiplied to obtain the final intactness.

### Device fabrication and electrical measurements

To probe electronic quality and exclude the interference from substrates, monolayer graphene after the transfer was encapsulated by two flakes of around 50 nm-thick hBN crystals. In detail, an hBN flake was picked up at 52 °C by a PPC/polydimethylsiloxane (PDMS) stack on a glass slide, which was attached to a micromanipulator. The as-formed hBN/PPC/PDMS stack was then used to pick up the graphene from the SiO_2_/Si at 52 °C. The “pick up” is possible because the van der Waals forces between hBN and the graphene are relatively stronger than that between SiO_2_/Si and the graphene. Subsequently, the graphene/hBN/PPC/PDMS stack was brought into contact with another hBN flake by releasing graphene/hBN at 70 °C from PPC surfaces, resulting the formation of the final hBN/graphene/hBN heterostructure. Electron-beam lithography and reactive ion etching (RIE) were employed to pattern the stack into a Hall bar geometry. After the RIE etching, Cr/Au (3/50 nm) electrodes were deposited by electron-beam evaporation for forming one-dimensional contacts.

The electrical properties of the fabricated devices were characterized by the conventional lock-in technique. An AC current *I*_ds_ with a root mean square amplitude of 1 μA at 23.33 Hz was applied between the source and drain terminals. Meanwhile, the four-point longitude voltage drop *V*_xx_ and transverse voltage drop *V*_xy_ were measured with lock-in amplifiers. The charge density tuning in the graphene channel is achieved by applying different back gate voltage (*V*_bg_). The device was tested in Argon inertial environment (glovebox at the temperature of 300 K) and vacuum environment (cryostat at a temperature lower than 100 K). *R*_xx_ is the longitude resistance, which can be obtained according to the equation: *R*_xx_ = *V*_xx_/*I*_ds_, and *R*_xy_ is the Hall resistance which can be obtained by *R*_xy_ = *V*_xy_/*I*_ds_. The longitude resistivity (*ρ*_xx_) can be calculated from *ρ*_xx_ = *R*_xx_ × W/L, where W is the width of the conducting channel, L is the length of the channel between the probed contacts. The longitude conductivity (*σ*_xx_) can be obtained via *σ*_xx_ = 1/*ρ*_xx_, while the Hall carrier density (*n*) can be determined by equation: *n* = *B*/(*eR*_xy_), where *e* is the elementary charge. Based on the Drude model, mobility (*µ*) can be estimated from the linear regions in the transfer curve (Fig. 4a) according to the equation: *µ* *=* *σ*_xx_/(*n*e).

Hall bar devices were fabricated on the graphene/SiO_2_/Si with marks for alignment. each graphene sample was etched into a Hall bar geometry using a PMMA etching mask (PMMA 950 K A4 @ 4000 rpm) designed by electron-beam lithography (EBL) (Raith 150 2nd) and plasma etching with air (Diener Pico). After using EBL to design a PMMA mask, Cr/Au (10/40 nm) electrodes were deposited on the samples using an electron-beam evaporator (Angstrom Engineering nexdep) and then a standard metal lift-off technique. The arrays of Hall bar devices on 4-inch transferred graphene were fabricated by maskless laser lithography system (Heidelberg MLA-150) with a photoresist (AR-P5350, Allresist EN). Electrical characterization at room temperature was performed in a vacuum probe station (Lakeshore CRX-VF) with a semiconductor characterization system (B1500A, KeySight).

### Graphene characterization

Raman spectra were obtained with Horiba LabRAM HR-800 with 532 nm and 633 nm laser. AFM (Bruker dimension icon) was used to characterize the morphology of the graphene samples. The element analysis was performed by XPS (Kratos Analytical AXIS-Ultra with monochromatic Al Kα X-ray). The roughness of as-transferred graphene and the thickness of OVMs and Polymer films were measured by using the white light interferometer (BW-S501). The optical transmittance of graphene was measured using a UV-VIS-NIR Spetrometer (Perkin-Elmer Lambda 950)

### Theoretical calculation

A series of MD simulations were performed to calculate the adhesion force through LAMMPS package. The schematic diagram is shown in Fig. 3c, in which a coarse-grained SiO_2_/Si layer contacts with SiO_2_-supported graphene. The top SiO_2_ layer approaches the supported graphene, and the adhesion stress could be obtained at different separation distances. The coarse-grained model of the SiO_2_ layers and the SiO_2_/Si substrates was used according to the literature. The interaction between the carbon atoms and the coarse-grained SiO_2_ particles was presented by Lennard-Jones potential $U\left(r\right)=4\varepsilon \left[{\left(\frac{{r}_{0}}{r}\right)}^{12}-{\left(\frac{{r}_{0}}{r}\right)}^{6}\right]$, with $\varepsilon =4.148{meV}$ and ${r}_{0}=2.79\mathring{\rm A}$. The AIREBO potential was employed to describe carbon-carbon interaction. During the simulation, SiO_2_ particles of the substrate were constrained to maintain its surface corrugations. The geometrical parameters of the substrates and the graphene sheets were obtained from the AFM images of bare substrates and supported graphene on substrates. In the case of graphene on PET, there is no reliable potential for calculating the van der Waals interaction. Therefore, we approximated the van der Waals interaction by adding a multiplier $\alpha$ to $\varepsilon$ in the above Lennard-Jones potential. We have simulated the situations of $\alpha =$ 0.5, 1, 2, and 3.

## Supplementary information

Supplementary information

## Data Availability

The data that support the findings of this study are available within the article and its Supplementary Information files. The source data underlying Figs.1d, e, 2c, 3b, d, 4a–f, 5a, c, and Supplementary Figs. 2c, e, g, 7i, 8a–i, 9f, 10a–d, 11a, b, 12a–c, and 13d–f are provided as “Source Data File”. All raw data generated during the current study are available from the corresponding authors upon request. Source data are provided with this paper.

## Source data

Source Data
