# Supplementary material for: Large-area transfer of two-dimensional materials free of cracks, contamination and wrinkles via controllable conformal contact
Source: Nat Commun. 2022 Jul 29;13:4409. doi: 10.1038/s41467-022-31887-z (PMC9338253; doi:10.1038/s41467-022-31887-z)
Supplement: Supplementary file 1 — Supplementary information [file 41467_2022_31887_MOESM1_ESM.pdf]

Supplementary information for:

Large-area transfer of two-dimensional materials free of cracks, contamination and wrinkles via controllable conformal contact

Yixuan Zhao[1#], Yuqing Song[1,2#], Zhaoning Hu[2#], Wendong Wang[3#], Zhenghua Chang[4,5], Yan Zhang[2], Qi Lu[6,2], Haotian Wu[2], Junhao Liao[7,8], Wentao Zou[2], Xin Gao[1,7], Kaicheng Jia[1], La Zhuo[2], Jingyi Hu[7], Qin Xie[7], Rui Zhang[3], Xiaorui Wang[2], Luzhao Sun[2], Fangfang Li[2], Liming Zheng[1], Ming Wang[2], Jiawei Yang[9,2], Boyang Mao[3], Tiantian Fang[10], Fuyi Wang[10], Haotian Zhong[2], Wenlin Liu[1], Rui Yan[2], Jianbo Yin[2], Yanfeng Zhang[11], Yujie Wei[4,5]*, Hailin Peng[1,2,7]*, Li Lin[11]*, Zhongfan Liu[1,2,7]*

Correspondence to: zfliu@pku.edu.cn; linli-cnc@pku.edu.cn; hlpeng@pku.edu.cn; yujie_wei@lnm.imech.ac.cn

This supplementary information includes:

Supplementary Figure 1-13

Supplementary Table 1-2

Reference (1-32)

Supplementary Figure 1: Illustration of crack formation after the removal of supporting films

For graphene lamination onto target substrates, such as SiO_2/Si, if the contact between graphene and target substrates was non-conformal, the presence of air gaps between graphene and the target substrates would turn the graphene free-standing at some regions. When we mechanically peel off or dissolve the supporting films, suspended regions are prone to be teared by the interfacial forces, which is caused by the mechanical delamination or the evaporation of organic solvent (Supplementary Fig. 1a). Therefore, if the conformal contact is formed, the substrate would also serve as the supporting substrates to undertake the interfacial forces, thereby avoiding cracks (Supplementary Fig. 1b)

The typical atomic force microscopy (AFM) images of graphene grown on Cu foils and Cu wafers were presented in Supplementary Fig. 1c-d**,** exhibiting that the presence of dense Cu steps contribute to the complex topographies of the as-grown graphene. After the coating of supporting films and the separation of graphene from Cu substrates, the graphene and supporting films would preserve these structures of underlying Cu. Supplementary Fig. 1e-f displays the typical AFM images of target substrates, SiO_2/Si and polyethylene terephthalate (PET) substrates with roughness that can be described by corrugated surfaces with a wavelength (35.3 nm for PET, 7.8 nm for SiO_2/Si) and an amplitude (1.4 nm for PET substrate, 184 pm for SiO_2/Si substrates). These values would be used in the following calculation of the adhesion energies of graphene with the substrates. Clearly, the roughness of both the graphene and substrates contribute to the difficulty in the formation of conformal contact.

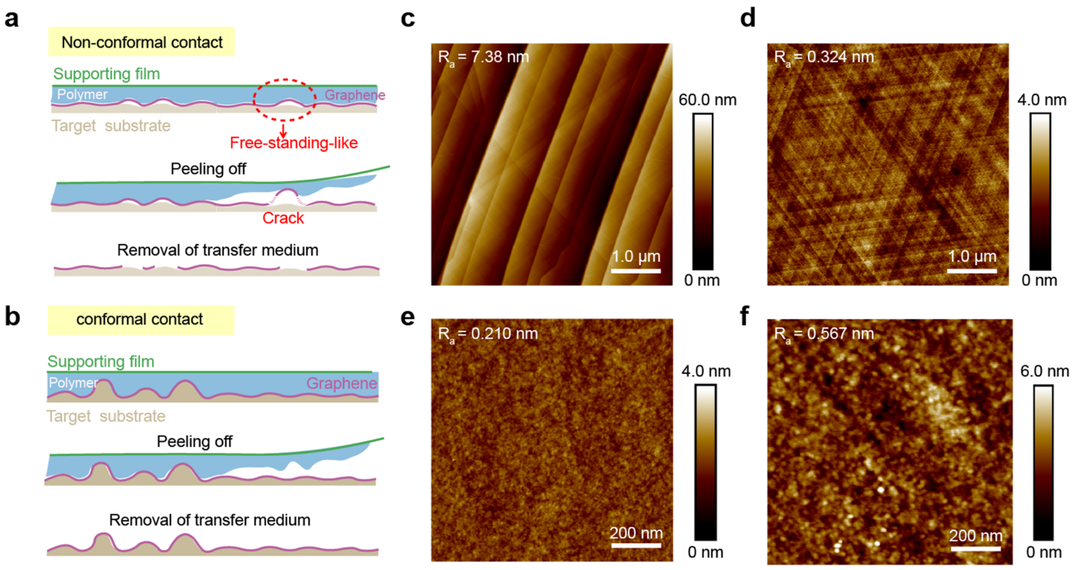

Supplementary Figure 1. Illustration of crack formation after the removal of supporting films. a, Illustration of crack formation in free-standing-like graphene regions after the removal of supporting films when the contact between graphene and substrates is non-conformal. **b**, Illustration of transferring graphene onto destination substrates free of cracks and contamination when the contact between graphene and substrates is conformal. **c-d**, Typical AFM images of CVD-grown graphene surfaces on commercially available Cu foil (**c**) and single-crystal Cu wafer (**d**). **e-f**, Typical AFM images of SiO_2/Si substrates (**e**) and PET substrates (**f**).

Supplementary Figure 2: Heat-induced height change of oxhydryl groups-containing volatile molecules (OVM)-modified PMMA and PPC/PMMA supporting films and its mechanism

The layer-by-layer blade coating of PPC and PMMA, both dissolved in anisole, onto graphene/Cu foils would form a composite film. Although both PPC and PMMA can be dissolved in anisole, after the evaporation of anisole, PMMA chains cannot be completely blended with PPC chains owing to the low miscibility of PMMA with PPC[1,2], and the movement of PPC chains is thus confined. In this regard, melt-blended approach has been used to form polymer blends[3]. PPC is amorphous with very low glass transition temperature (T_g) (20-40 ºC). Therefore, upon heating, owing to the low T_g, some PPC chains start to relatively move in a viscous state, resulting in the adequate blending of PPC with PMMA chains, which smoothed out the rough surface of graphene. The adequate blending of polymer chains would therefore induce the observed height reduction of the entire films by restacking of polymer chains. In addition, above T_g, the composite polymer in a viscous state would be prone to spread over a rough surface, also contributing to the complete conformal contact between graphene and PET substrates. These altogether contribute to the conformal contact between graphene and substrate (Supplementary Fig. 2a).

As discussed above and in the main text, under heat, the deformation of supporting films would be induced by the evaporation of OVMs embedded in PMMA chains or the glass transition of PPC in PMMA. The observed height change of the supporting films in white light interference images under heat can provide the direct evidence of the deformation. In this regard, we only coated the modified supporting films on SiO_2/Si substrates and monitored the heat-induced deformation. To measure the height change, we *in-situ* measured the difference in height between the bare substrates and the supporting films at the edge of films before and after the heating. After heating (same conditions for transferring graphene), a larger height change was observed for the OVMs-modified PMMA (Supplementary Fig. 2b-c) and for the PPC/PMMA films

(Supplementary Fig. 2d-e) in white light interference images. In contrast, for pure PMMA, a very small height change can be induced by the heat treatment (Supplementary Fig. 2f-g). In addition, the height change can be tuned by the amount of OVMs in the OVMs-modified supporting films (Supplementary Fig. 2h).

In our work, PPC/PMMA was used to transfer graphene grown on rough Cu foil onto rough PET substrates, and OVMs-modified PMMA was used to transfer graphene grown on flat Cu wafer onto relatively flat SiO_2/Si substrates. From the view of application, inch-sized graphene single crystals grown on ultraflat Cu (111) wafer with low density of wrinkles and defects would be potentially targeted for the applications in electronics and optoelectronics, which require the graphene sample on silicon-based substrate, while graphene grown on relatively roughness Cu surface with fine scalability would be suitable for future wearable and flexible electronics, in which transfer of graphene onto flexible substrates would be preferred, such as PET.

When transferring graphene onto flat SiO_2/Si substrates, the adhesion between graphene and substrate is relatively weak than that between graphene and PET. Although the roughness of graphene inherited from the Cu foils would be reduced when using PPC/PMMA, the relatively strong interaction between PPC and graphene would peel some graphene flakes off the substrate when the polymer is directly peeled off from the graphene surface. Therefore, the PPC/PMMA would not be suitable for the transfer of graphene onto silicon substrates.

When transferring graphene onto PET substrates, OVMs-modified PMMA is not suitable for transferring graphene onto PET substrates due to the rough surface of PET. Owing to the low glassy transition temperature of PPC, upon heating, the height change of PPC/PMMA caused by the glassy transition of PPC would flatten the graphene surface, and the PPC would turn into liquid-like state and push the graphene films onto the rough PET substrates conformally, ensuring the successful transfer of graphene onto PET substrates.

Thus, we believe the roughness of target substrates and the interaction between graphene and polymer would be the main concerns that determine the choice of transfer medium.

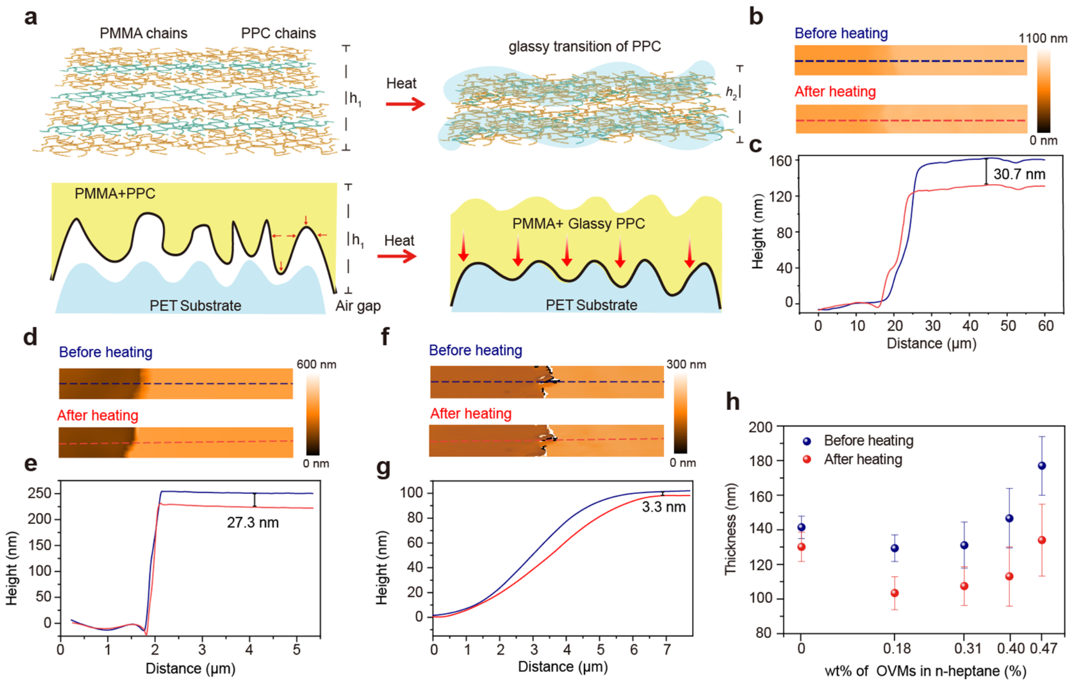

Supplementary Figure 2. Heat-induced height change of OVMs-modified PMMA and PPC/PMMA supporting films. a, Mechanism illustration of the heat-induced deformation and resulted conformal contact using PPC/PMMA as supporting films. Heat treatment would incur the height change from h1 to h2. **b**, White light interference images of OVMs-modified supporting films edge before (navy blue) and after (red) the *in-situ* heat treatment. **c**, The corresponding height along the dash line in the white light interference images in (**b**) before (navy blue) and after (red) heat treatment. **d**, White light interference images of PPC/PMMA supporting films edge before (navy blue) and after (red) the *in-situ* heat treatment. **e**, The corresponding height along the dash line in the white light interference images in (**d**) before (navy blue) and after (red) heat treatment. **f**, White light interference images of PMMA-only supporting films edge before (navy blue) and after (red) the *in-situ* heat treatment. **g**, The corresponding height along the dash line in the white light interference images in (**f**) before (navy blue) and after (red) heat treatment. **h**, The obtained height before (navy blue) and after (red) the

heat treatment from the white light interference images as function of OVMs concentration. The error bars depict the standard deviation of the thickness.

Supplementary Figure 3: Transfer of graphene on SiO_2/Si substrates by PMMA-based transfer assisted by TRT, conventional PMMA-based method, and OVMs-modified supporting films.

The unsuccessful lamination of graphene onto target substrates and the poor conformity between graphene and substrates would result in the formation of cracks during the transfer. For large-area graphene transfer by PMMA-based transfer assisted by TRT, the high flexibility of PMMA membrane would result in the formation of centimetre-scale folds and wrinkles during the bubbling-based delamination, and it is also difficult to rinse and dry the PMMA/graphene membrane. Therefore, we used the thermal release tape (TRT)[4] as the rigid supporting layer to enhance large-area operability in the transfer of graphene with PMMA. In detail, we first coated the PMMA on 4-inch-sized graphene single crystals/Cu wafer, and laminated the TRT onto PMMA after the curing of PMMA. Subsequently, bubbling-based delamination of graphene from Cu was conducted, followed by rinsing and drying the TRT/PMMA/graphene membrane. Then we laminated graphene onto target substrates in a dry environment. After the lamination, heat treatment of the TRT would significantly reduce the adhesion energy between TRT and PMMA, which would leave the PMMA/graphene onto the target substrates. However, we usually found that the films cannot be entirely released from TRT owing to the unsuccessful lamination of graphene onto the substrates with weak interactions between graphene and substrates. This would result in the formation of centimetre-sized cracks (Supplementary Fig. 3a). Note that, even only TRT was used as the supporting films without PMMA, centimeter-sized unsuccessful lamination still occurs especially for the dry lamination. Unsuccessful lamination is usually caused by the non-uniform interfacial force and poor contacting between graphene and substrates, and large bubbles are usually trapped during the dynamic lamination. Similarly, nanoscale or microscale incomplete conformal contact would result in the formation of small air bubbles between graphene and substrates, as already observed in the fabrication of van der Waals heterostructures[5]. The region in presence of air bubbles

would become nearly free-standing and be prone to being teared after the dissolving of PMMA (Supplementary Fig. 3b-c).

As for conventional PMMA-based transfer, the incomplete removal of PMMA by acetone leaves the polymer residues on graphene surface (Supplementary Fig. 3d-e). Furthermore, the trapped water between graphene and substrates results in the difficulty in forming the conformal contact between graphene and SiO_2/Si, which would cause the formation of wrinkles and cracks in the conventional PMMA-based transfer, as evidenced by the AFM images and OM images (Supplementary Fig. 3d, f).

The oxhydryl groups-containing volatile molecules (OVM) including cedrol, alpha-terpineol, linalool and (-)-borneol, all have oxhydryl groups that can form a strong hydrogen bond with the carbonyl oxygen in the PMMA chains (Supplementary Fig. 3g). All these molecules are volatile, and therefore can evaporate under heat, resulting in the restacking of PMMA chains and the observed deformation of supporting films. During the hot lamination of graphene onto substrates, such deformation would finally enable the formation of conformal contact between graphene and target substrates, key for the crack-free transfer. The crack-free transfer was confirmed by the uniform contrast in the photographs with 12-megapixel resolution capable of visualizing the centimeter-sized cracks (Supplementary Fig. 3h) and optical microscopy (OM) images with 5× and 50× magnifications that can be used to characterize micrometer-sized cracks (Supplementary Fig. 3i). Note that the choice of OVMs would not influence the intactness of transferred graphene. It is the volatile nature and oxhydryl group in OVMs that are the key for the successful transfer.

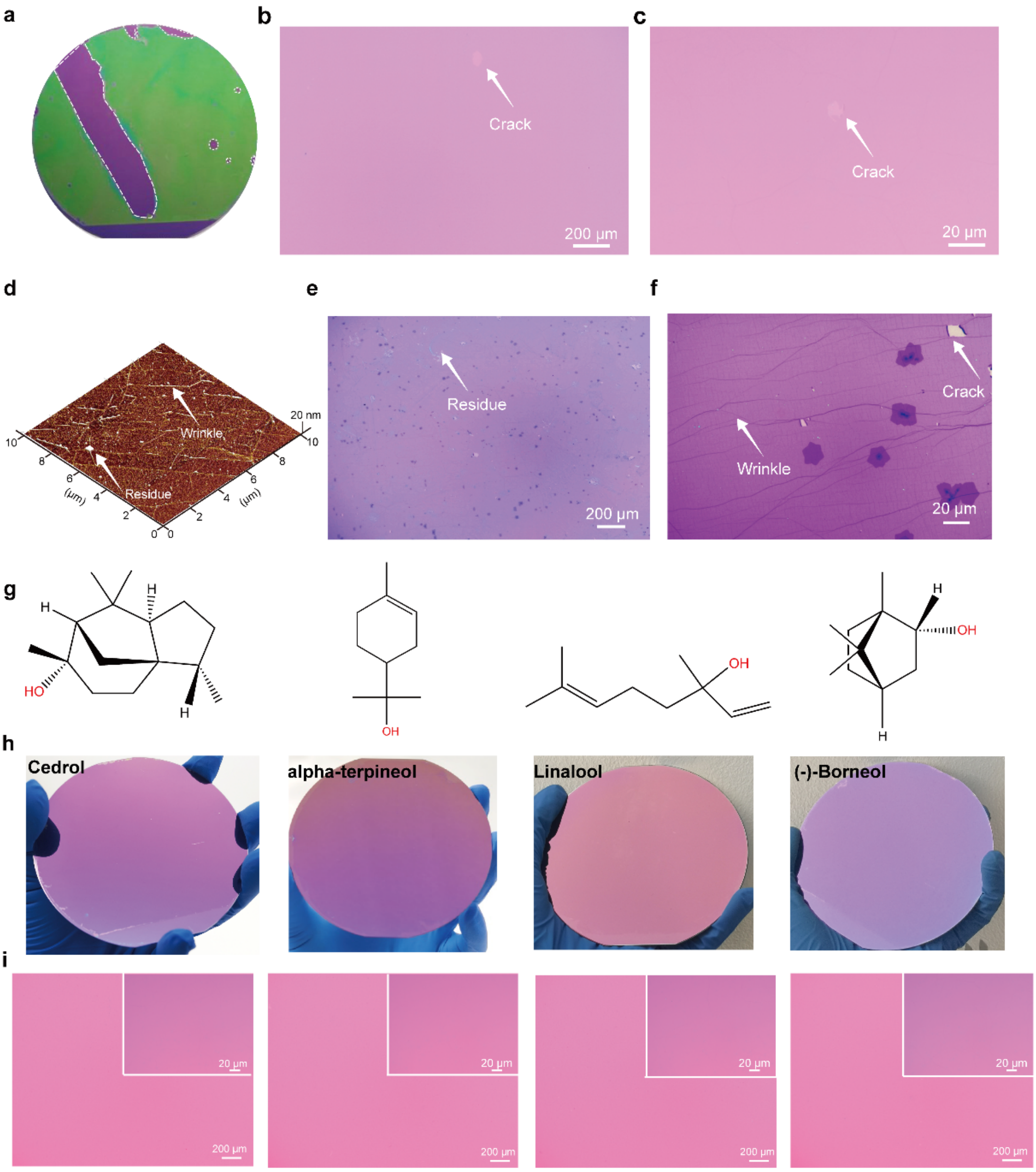

Supplementary Figure 3. Transfer of graphene on SiO_2/Si substrates by PMMA-based transfer assisted by TRT, conventional PMMA-based method and OVMs-modified supporting films. a-c, PMMA-based transfer assisted by TRT. Photograph of the 4-inch-sized graphene transferred on SiO_2/Si substrates with the assistance of PMMA. Cracks of the PMMA membrane are denoted by white dash lines (**a**). OM images of as-transferred graphene by PMMA with 5× (**b**) and 50× (**c**) magnifications. Note that the location of cracks was highlighted by white arrows. **d-f**, Conventional PMMA-based method of transferring graphene onto SiO_2/Si. Typical AFM images of

as-transferred graphene (**d**). OM images of as-transferred graphene with 5× (**e**) and 50× (**f**) magnifications. Note that the locations of cracks/residues/wrinkles were highlighted by white arrows. **g**, The structural formula of cedrol, alpha-terpineol, linalool and (-)-borneol. **h**, Photographs of the as-transferred 4-inch-sized graphene single-crystal wafer on SiO_2/Si substrates by OVMs-modified supporting films. The intactness of as-transferred graphene by cedrol, alpha-terpineol, linalool and (-)-borneol is 99.6%, 99.0%, 99.3% and 99.5% respectively. **i**, Corresponding OM images of the graphene transferred by OVMs-modified supporting films with 5× and 50× magnifications (inset).

Supplementary Figure 4: Intactness characterization of graphene on PET substrates transferred by conventional method and our design.

We also observed the formation of centimetre-sized and micrometre-sized cracks (Supplementary Fig. 4a-c) after the transfer of A4-sized graphene film onto PET substrate using the conventional PMMA-based method. Note that, large-area visualization of cracks on PET substrates were done by using a commercial scanner (24-megapixel resolution), because the reflection mode can enhance the contrast difference between regions covered and uncovered by graphene. Similarly, by adding PPC to enable conformal contact, almost no cracks would be formed, as evidenced by uniform contrast in A4-sized scanning image (Supplementary Fig. 4d) and OM images with 5× and 50× magnifications (Supplementary Fig. 4e-j).

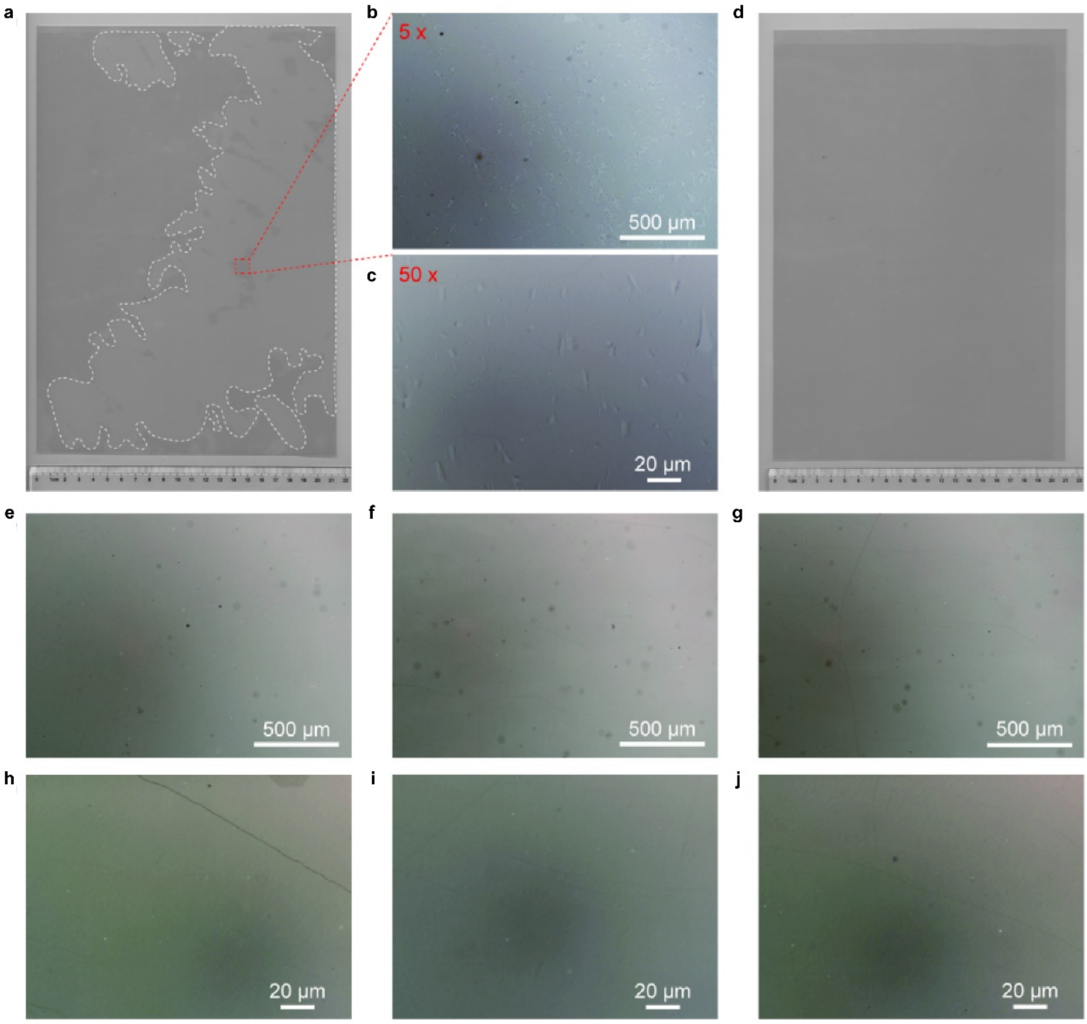

Supplementary Figure 4. Intactness characterization of graphene on PET substrates transferred by conventional method and our design. a-**c**, Scanned image (**a**) and OM images (**b-c**) of A4-sized graphene films transferred onto PET substrates by PMMA-only supporting films. **d**, Scanned image of the as-transferred A4-sized graphene on PET substrates by PPC/PMMA supporting films. **e-j**, Typical OM images of the as-transferred graphene with 5× (**e-g**) and 50× (**h-j**) magnifications onto PET substrates.

Supplementary Figure 5: The reliability of characterizing the micrometer-sized cracks using OM images with 5× and 50× magnifications

OM can be used to characterize the micrometer-sized cracks, and the available magnifications usually include 5×, 10×, 20×, 50× and 100×. In our case, the reliability with high sampling representativeness of the obtained values would determine the choice of magnifications. Large-area characterization would ensure the high sampling representativeness. To attain this, we first took the OM images with 5× magnification to characterize intactness over large scale, and we found that 5× magnification is capable of visualizing cracks larger than 7 μm (Supplementary Fig. 5a-e). Therefore, OM images with 50× and 100× magnifications should be used to visualize the smaller cracks. 50× magnification can characterize the larger area, while 100× magnification can visualize the very small cracks (~200 nm according to the resolution limitation of OM). It was found that the observed smallest cracks are usually around 1 μm, still visible in OM image with 50× magnification. Therefore, the 50× magnification that can characterize larger region would ensure a higher sampling representativeness, as also confirmed by the narrower distribution of inferred intactness based on 50× magnification OM images than that with 100× magnification (Supplementary Fig. 5f). Therefore, OM images were taken at 5× and 50× magnifications to comprehensively characterize the micrometer-sized cracks for graphene transferred onto both SiO_2/Si and PET substrates.

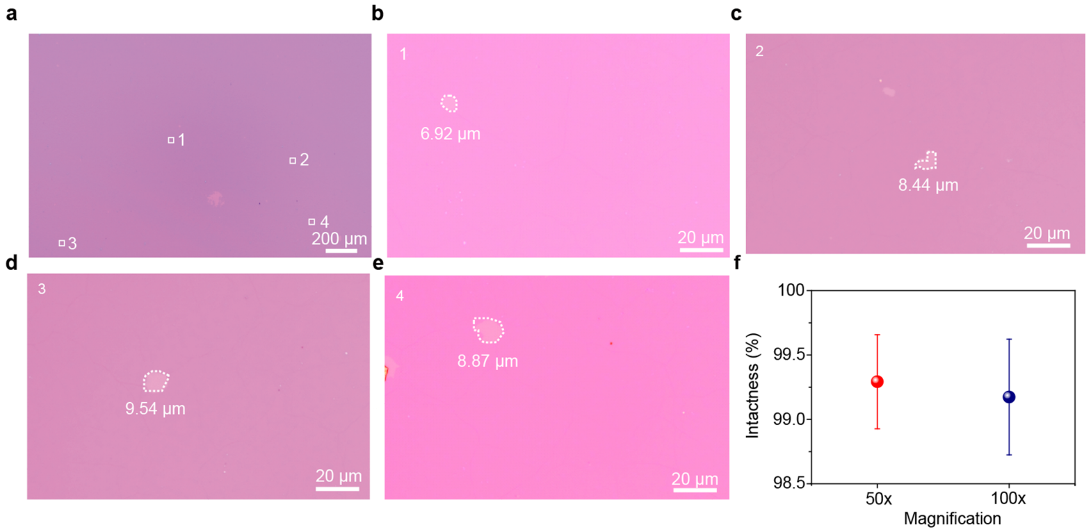

Supplementary Figure 5. The reliability of characterizing the micrometer-sized cracks using OM image with 5× and 50× magnifications. a-e, OM images of graphene on SiO_2/Si substrates with the micrometer-sized cracks. The size of corresponding cracks in (**a**) was checked by OM image with 50× magnification (**b-e**). **f**, Statistical distribution of intactness based on OM images of the same sample with 50× (red) and 100× (blue) magnifications. The graphene was transferred on SiO_2/Si substrates by using cedrol (10 wt%)/PMMA as OVMs-modified supporting films. The error bars depict the standard deviation of the intactness.

Supplementary Figure 6: Customized bubbling-delamination equipment with controllable delamination rate and force

Cost-efficient electrochemical bubbling-based delamination process that would enable the recycling of the growth substrates requires the generation of rich hydrogen bubbles in the negative electrode which can intercalate between graphene and Cu[6]. Therefore, graphene on Cu foils would function as the negative electrode, and we found that by controlling the position of positive electrode (platinum electrode used for the delamination of A4-sized graphene from Cu foils), we can control the position where the delamination occurs with the highest rate. This is crucial to avoid cracks especially in large-area transfer, since the nonuniform delamination would also result in the cracks formation. To attain this, we designed customized bubbling-delamination equipment for transferring A4-sized graphene films, in which graphene/Cu is connected to the negative electrode. The platinum electrode as the positive electrode is mobile along the Cu foil, and was placed near graphene/Cu foil to enhance electric fields and to form rich hydrogen bubbles (Supplementary Fig. 6a-b). To enhance the delamination rates, a mechanical force was applied to the supporting layer to assist the peeling of graphene off Cu foils. A stepper motor can be used to control the peeling rate. The delamination rate would be balanced by the moving rate of platinum electrode and the peeling rate of graphene. In addition, the Cu foil was fixed on an auxiliary fixture to further ensure a uniform delamination force.

In the delamination of graphene from Cu wafers, we designed a customized transfer equipment which can achieve the delamination of four 4-inch-sized graphene single-crystal wafers in one batch together. Graphene on Cu wafers were connected to the negative electrode, while fixed graphite plate was used as the positive electrode (Supplementary Fig. 6c-d). A mechanical force was applied to achieve the peeling of graphene/supporting films off Cu wafers: the delamination rate was enhanced through a separation bar, and the separation bar was linked to two stepper motors which can control the force and separation speed in two directions, as denoted. The whole

equipment includes a cleaning unit. After the separation process, graphene wafers and Cu wafers were rinsed with deionized water in a cleaning unit that comprises 5 water tanks. Finally, both graphene on supporting films and Cu wafers would be dried by nitrogen gas, and Cu wafers can be used for the regrowth of graphene.

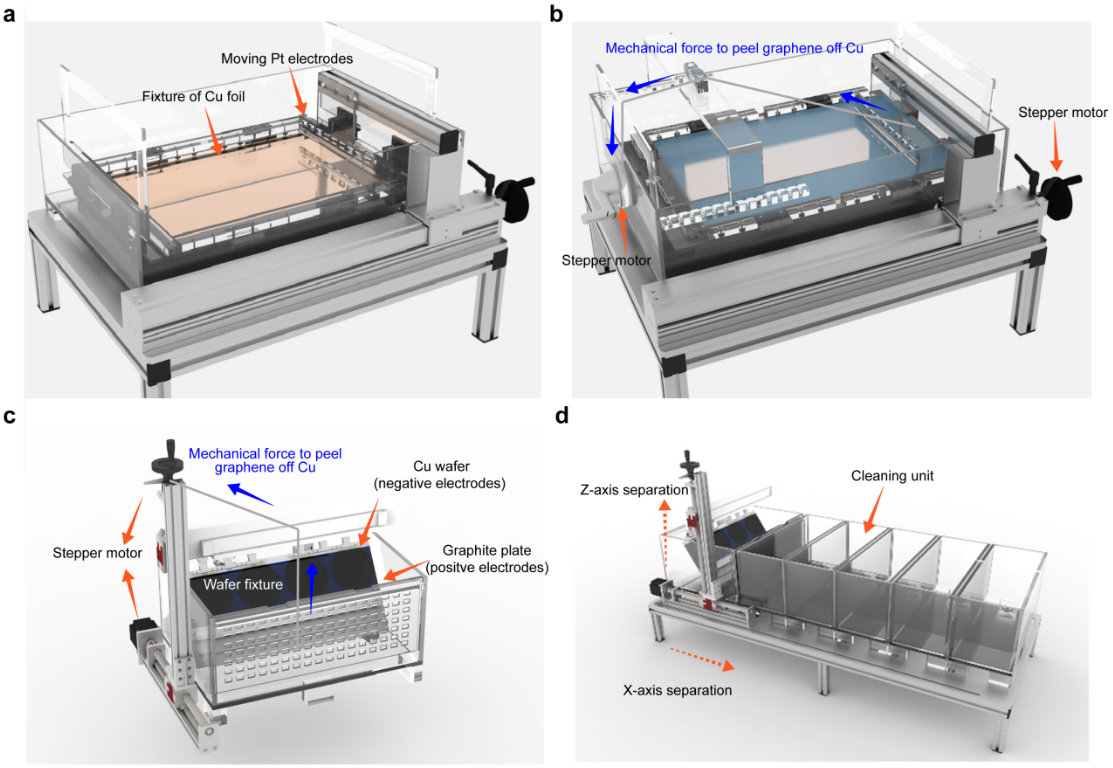

Supplementary Figure 6. Customized bubbling-delamination equipment with controllable delamination rate and force. Schematic illustration of customized equipment for the bubbling-based delamination of graphene from Cu foils (**a-b**) and Cu wafers (**c-d**).

Supplementary Figure 7: Roughness of the whole 4-inch graphene wafer

In order to characterize the roughness of whole 4-inch graphene wafer, AFM images with the size of 25 × 25 μm were taken as shown in Supplementary Fig. 7a-h, in which the AFM results confirm the residue-free transfer of the graphene. In addition, by using white light interferometer, a comprehensive investigation of the roughness was conducted over the entire 4-inch sized wafer: we have measured the roughness of 50 representative areas at the top, bottom, middle, right and left region of the wafer. As shown in Supplementary Fig. 7i, the as-obtained roughness is 0.25 ± 0.03 nm, consistent with the AFM results.

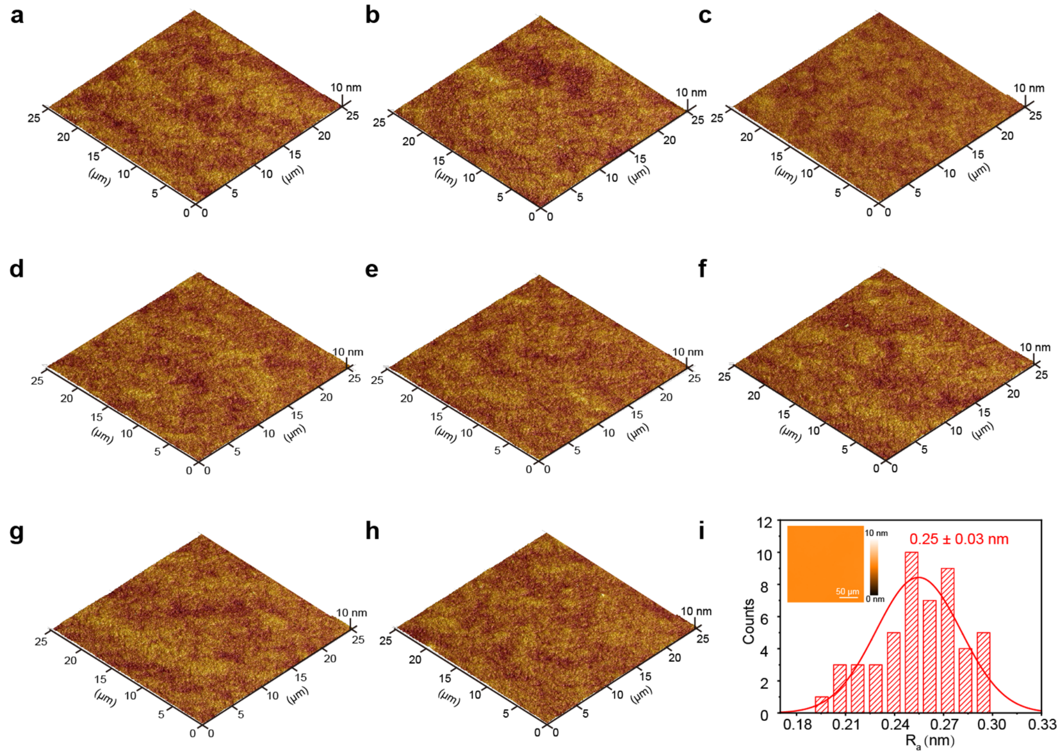

Supplementary Figure 7. Roughness of the whole 4-inch graphene wafer. a-h, Typical AFM images of as-transferred graphene on SiO_2/Si substrates with 25×25 μm across the 4-inch graphene wafer. **i**, Statistical distribution of graphene roughness on SiO_2/Si substrates obtained by white light interferometer. Inset: corresponding white

light interference images. Note that graphene above was transferred on SiO_2/Si substrates by alpha-terpineol (10 wt%)/PMMA.

Supplementary Figure 8: Contamination- and wrinkle-free transfer of graphene onto SiO_2/Si and PET substrates

AFM imaging was conducted to probe the contamination on graphene surface. In this regard, the clean graphene surface on SiO_2/Si substrates by mechanically peeling the supporting films off graphene surface was confirmed by the uniform contrast in corresponding AFM image (Fig.2b, main text). Especially, with average roughness (R_a) of 0.183 nm, the corresponding height distribution of resulted surface is similar to that of bare SiO_2/Si substrates (Supplementary Fig. 8a) which means that the observed roughness in clean graphene surface is caused by the substrate roughness. In clear contrast, owing to the presence of few-layer contamination, a broader peak in height distribution was observed for the unclean surface (transferred with conventional PMMA-based method) (Supplementary Fig. 8b). In addition, a side peak was caused by the presence of higher residue particles.

Large-area characterization of contamination (polymer residues) concentration was conducted by using deuterium-labeled PMMA in the transfer of graphene. In time-of-flight secondary ion mass spectroscopy (ToF-SIMS), the peak intensity of deuterium in PMMA can be used to reflect the concentration of polymer residues[7]. Clearly, the peak intensity of deuterium was highly reduced when using the PPC/PMMA as the supporting films to transfer A4-sized graphene from Cu foils onto PET substrates (Supplementary Fig. 8c), consistent with the results of transferred graphene on SiO_2/Si substrates (Fig. 2c, main text). Note that the amount of deuterium-labeled PMMA was kept same for different transfers. Therefore, the direct peeling of supporting films from graphene enabled by PPC/PMMA significantly reduced the polymer residues.

In addition, the quantification of PMMA, TRT and OVMs residues on the surface of transferred graphene can be obtained by XPS results[8,9]. We coated PMMA, TRT and OVMs on SiO_2/Si and obtained the corresponding C1*s* spectrum peaks (Supplementary Fig. 8d-f). We also probe the cleanness of the as-transferred graphene by using our

method and conventional PMMA-based transfer (Supplementary Fig. 8g-h). Comparing the XPS results of TRT, as-transferred graphene, PMMA, and OVMs on SiO_2/Si substrates, it was clearly shown that the graphene transferred by our method (Supplementary Fig. 8h) exhibited no residue-related C-O and O-C=O peaks observed in XPS results of PMMA, TRT, and OVMs, and exhibited similar peak with that of bare substrates (Supplementary Fig. 8i), confirming the improved cleanness. In contrast, in PMMA-based transfer, the PMMA-induced peak was clearly visible in XPS spectrum (Supplementary Fig. 8g)

When graphene become free-standing, the formation of graphene wrinkles would be favorable to lower the system energy[10]. The conformal contact would avoid the presence of free-standing graphene after the removal of supporting films, and therefore the conformal state would suppress the formation of new wrinkles, as evidenced by the uniform contrast in the OM images of graphene transferred from ultraflat Cu wafers onto SiO_2/Si substrates (Supplementary Fig. 8j-l). Note that the wrinkle-free graphene can be grown on ultraflat Cu by suppressing the formation of wrinkles during the high-temperature CVD growth[11], and our approach can avoid the wrinkle formation during the transfer.

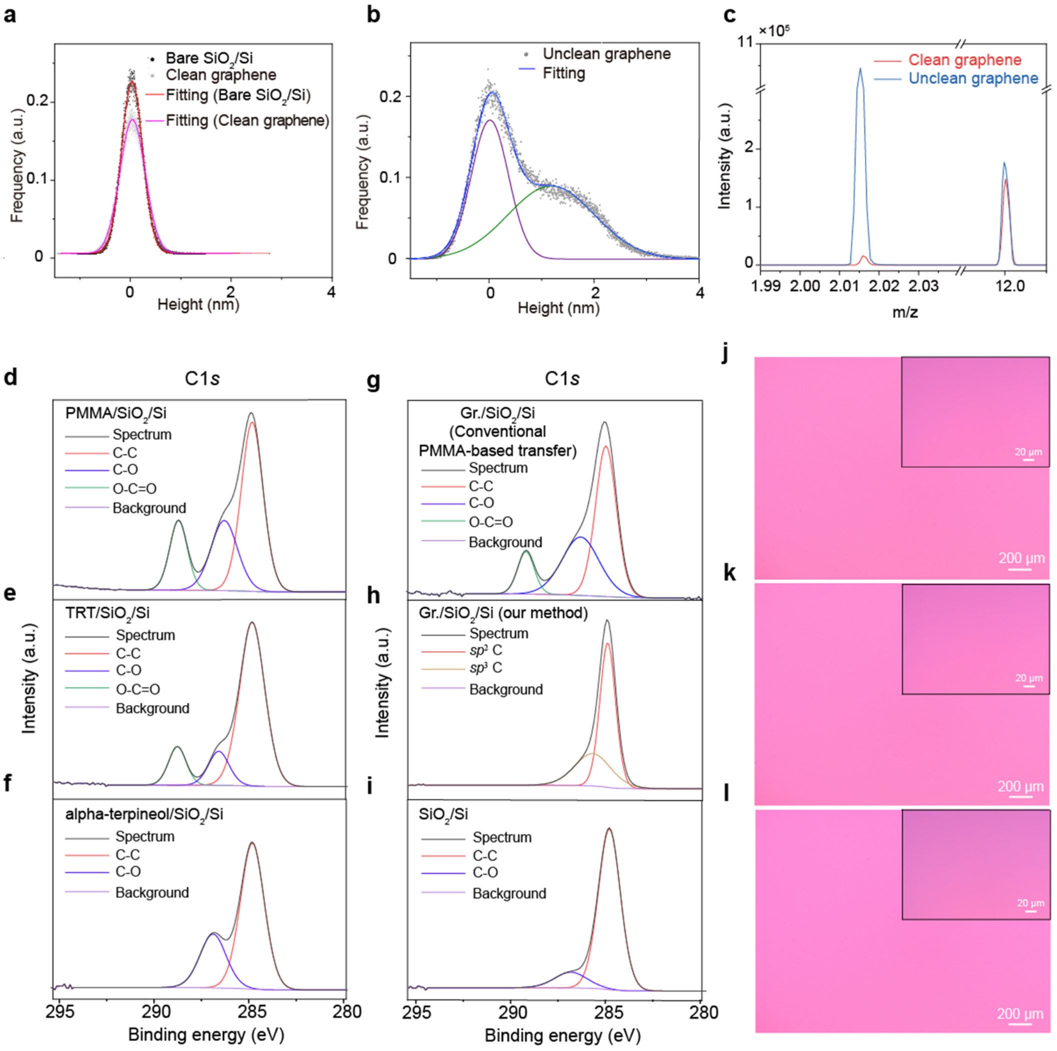

Supplementary Figure 8. Contamination- and wrinkle-free transfer of graphene onto SiO_2/Si and PET substrates. a, Corresponding height distribution of as-transferred graphene on SiO_2/Si substrates by OVMs-modified PMMA (purple) (cedrol (10 wt%)/PMMA) and bare SiO_2/Si (red). The height distribution was obtained from the AFM images in Fig. 2b and Supplementary Fig. 1e. **b**, Corresponding height distribution of as-transferred graphene on SiO_2/Si substrates by conventional PMMA-based techniques. The height distribution was obtained from the AFM image in Fig. 2a. The FWHMs of peaks in height distribution are 0.70 nm, 0.53 nm, 0.44 nm, for unclean graphene, clean graphene and bare substrate, respectively. The larger FWHM is caused by the presence of few-layer contamination. **c**, ToF-SIMS spectra of as-transferred graphene on PET substrates by conventional PMMA-based techniques (blue line) and our design (red line). Note that the peaks around 2 and 12 correspond to the $^2H^-$ and C^-

peak, respectively. **d-f,** C1*s* core level XPS spectra of PMMA (**d**), TRT (**e**) and OVMs (alpha-terpineol) (**f**). **g-i**, C1*s* core level XPS spectra of graphene transferred by conventional PMMA-base method (**g**) and our method (alpha-terpineol, 10 wt%) (**h**) as well as bare SiO_2/Si (**i**). Note that the peak of C-C should be composed of sp^2 and sp^3 carbon, which is hardly to be discriminated in our case. **j**-**l**, Typical OM images of graphene transferred onto SiO_2/Si with 5× and 50× magnifications (inset). Note that graphene in (**j**)-(**l**) was transferred on SiO_2/Si substrates by alpha-terpineol (10 wt%)/PMMA.

Supplementary Figure 9: The difference in height between monolayer graphene-covered substrates and SiO_2/Si substrates and the engineering of adhesion

The incomplete conformity between graphene and the substrate would produce air gaps full of oxygen and water molecules, which can be inferred from the larger difference in height between graphene-covered substrates and the bare substrates. To confirm that, we collected the AFM images of graphene edges after the transfer. As indicated in Supplementary Fig. 9a-d, the difference in height between transferred graphene using PMMA-only supporting films and substrate is clearly larger than that between transferred graphene using OVMs-modified supporting films and substrates. During the AFM imaging of graphene transferred by PMMA, we investigated the region without PMMA residues to exclude the interference from contamination. Note that the height fluctuation in the graphene-covered region (graphene itself) is almost negligible in comparison with that at the graphene edge. Therefore, it can be concluded that the incomplete conformity would produce larger gaps between graphene and substrates.

Evidenced by AFM images, the fine conformity of transferred graphene using OVMs-modified supporting films (Supplementary Fig. 9c-d) was similar with that of the exfoliated graphene on SiO_2/Si substrates (Supplementary Fig. 9e-f). The better conformity between exfoliated monolayer graphene and substrates is required to provide sufficient adhesion energy that ensures the successful exfoliation between layers. Importantly, inferred from previously reported AFM images of CVD graphene transferred onto SiO_2/Si substrates, the differences in height between graphene-covered substrates and bare substrates were also large, consistent with our observation (Supplementary Fig. 9h) [12-17].

For understanding the relationship between the conformity and adhesion energy, we calculated the stress-separation relationship between the graphene and substrates (Fig. 3c and Supplementary Fig. 9i). Since there are no available potentials for describing the van der Waals interaction between PET substrates and graphene, we conducted the

approximation based on the Lennard-Jones potential that was used to calculate interaction between SiO_2/Si and graphene (Fig. 3c, main text). In detail, we add a multiplier α to ε in the above Lennard-Jones potential to describe the graphene-PET interaction ($\alpha\varepsilon$ is the potential well depth for the graphene-PET interaction). We simulated the situations with $\alpha = 1/2,\ 1$, 2 and 3. As shown in Supplementary Fig. 9f, in all the four cases, the adhesion-separation relationships are still in the form of the Lennard-Jones potential, in which after the peak value, the adhesion would be rapidly reduced with increasing separation distance.

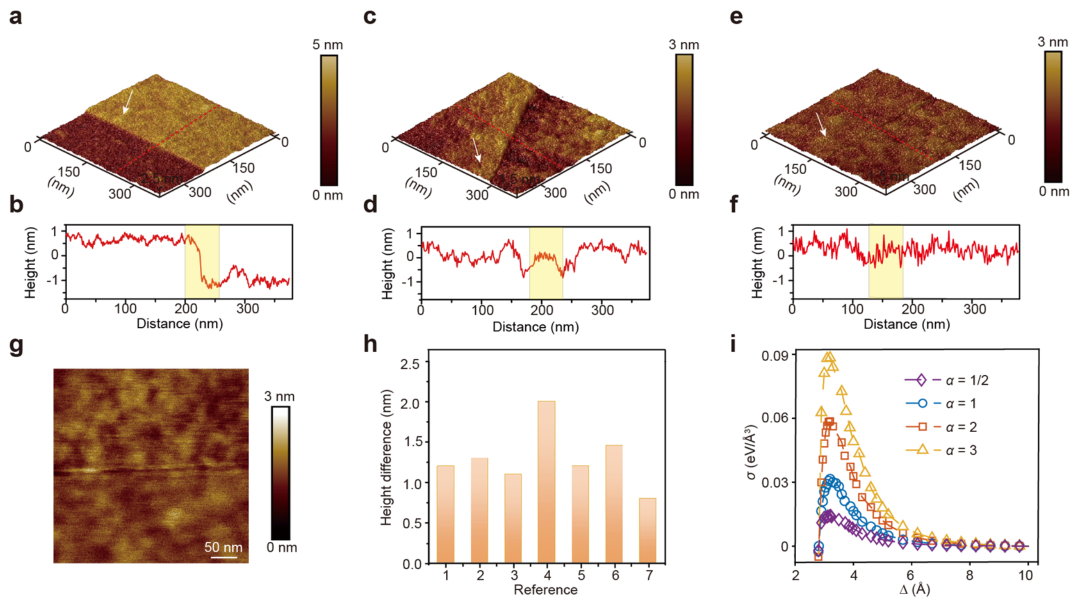

Supplementary Figure 9. The conformity-mediated engineering of adhesion between graphene and substrates. a-d, AFM images with 3D mode of graphene transferred by PMMA (**a**) and by OVMs-modified supporting films (cedrol (10 wt%)/PMMA) (**c**), and the height as a function of distance along the red dash line (b, d). The white arrow denotes the position of graphene edge. **e-g**, AFM images with 3D (**e**) and 2D (**g**) modes of mechanically exfoliated graphene on SiO_2/Si substrates, and the height as a function of distance along the red dash line (f). The white arrow denotes

the position of graphene edge. **h**, Statistics of the difference in height between monolayer graphene and the substrate inferred from or reported by previously published AFM images. **i**, Calculated stress-separation relationship between the PET and graphene. We approximated the van der Waals interaction by adding a multiplier α to ε in the Lennard-Jones potential. We have simulated the situations of α =0.5, 1, 2 and 3.

Supplementary Figure 10: Raman characterization of as-transferred graphene

The trapped water molecules would induce the *p*-doping effect, which would influence the intensity ratio of 2D band and G band as well as the full width at half maximum (FWHM) of the 2D band[16,17]. As shown in Supplementary Fig. 10a-b, the FWHM of the 2D band of graphene transferred onto SiO_2/Si substrates by OVMs-modified supporting film is around 24 cm^{-1}, comparable to the reported values of mechanically exfoliated graphene[18,19], confirming the suppressed *p*-doping effect by conformity. Furthermore, in our case, the intensity ratio of 2D band to G band is near 2, comparable to the mechanically exfoliated counterpart (Supplementary Fig. 10c)[20]. To exclude the substrate interference, we encapsulated the transferred graphene by hexagonal boron nitride (hBN). The FWHM of 2D band is further reduced to around 17 cm^{-1} (Fig. 4d in main text and Supplementary Fig. 10b), and the intensity ratio of 2D band to G band is near 8 (Supplementary Fig. 10c).

As shown in the Supplementary Fig.10d, the corresponding 2D peak position (ω_{2D}) as a function of G peak position (ω_G) was used to reflect the doping and strain level of graphene before and after the transfer[19]. Directly after the growth, graphene on Cu substrates (green dots in Supplementary Fig. 10d) suffered from strong compressive strain before transferring. The as-transferred graphene films by conventional PMMA-based method (blue dots in Supplementary Fig. 10d) suffered from tensile stress and were highly p-doped due to water doping at the interface, both of which would results in the reduced carrier mobility. In contrast, graphene transferred on SiO_2/Si substrates by our method experienced reduced strain and p-doping near the “intrinsic point” (purple dots in Supplementary Fig.10 d). Therefore, after the CVD growth, graphene is strongly compressively stressed, and such prestress might relatively be balanced by the formation of conformal contact, which contributes to the observed high carrier mobility of graphene. However, it should be admitted that there is still relatively little tensile strain in graphene, which might be caused by the conformal contact[12].

All above observation confirms the high quality of transferred graphene with reduced doping caused by the trapped oxygen and water molecules.

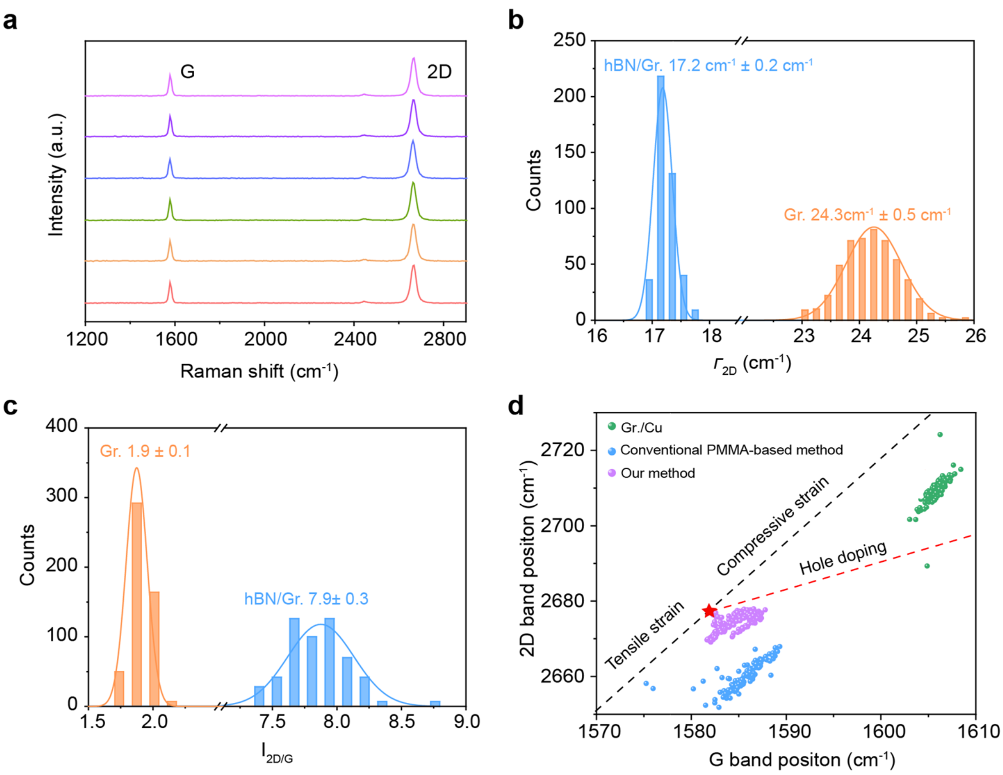

Supplementary Figure 10. Raman characterization of as-transferred graphene. a, Raman spectra of graphene transferred on SiO_2/Si substrates by OVMs-modified supporting films (alpha-terpineol (10 wt%)/PMMA). **b**, Statistical distribution of FWHM of graphene 2D band on SiO_2/Si substrates (orange) and encapsulated by hBN (blue). **c**, Statistical distribution of intensity ratio of Raman 2D band to G band of graphene on SiO_2/Si substrates (orange) and encapsulated by hBN (blue). **d**, The G peak positions as function of 2D peak positions of graphene on SiO_2/Si substrates transferred by conventional PMMA-based methods (blue dots), our methods (purple dots) (cedrol (10 wt%)/PMMA) and graphene on growth substrates measured directly after the growth (green dots). The red star represents the G and 2D peak positions of the pristine graphene with neither doping nor strain. The red dashed line is an average of experimental results for strain-free graphene doped by varying the density of holes. The

black dashed line represents a prediction of charge-neutral graphene under randomly oriented uniaxial stress[19].

Supplementary Figure 11: Electrical properties of transferred graphene

In order to probe carrier mobility of graphene on SiO_2/Si, we fabricated Hall bar devices with 1.2 cm interval over the entire 4-inch wafer, (inset: Supplementary Fig. 11b) and measured the field effect transistor (FET) carrier mobility (inset: Supplementary Fig. 11a). It was found that the average value of 18 devices is 8800 $cm^2 V^{-1} s^{-1}$ (Supplementary Fig. 11a, b), which is relatively higher than previous report[21-25].

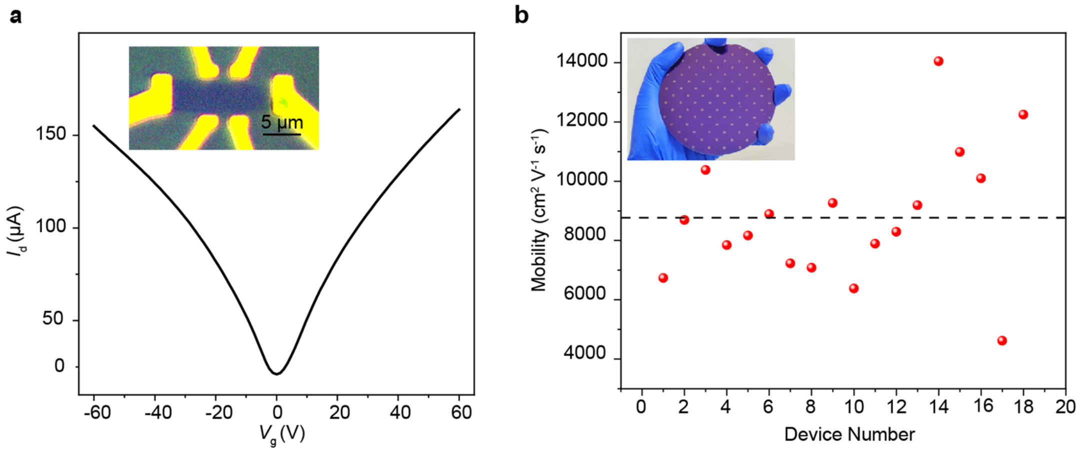

Supplementary Figure 11. Electrical properties of transferred graphene. a, Transfer characteristics of typical Hall bar devices fabricated with graphene transferred by OVMs-modified supporting films (alpha-terpineol (10 wt%)/PMMA). Inset: OM images of graphene Hall bar device on SiO_2/Si. **b**, Statistical results of the carrier mobility of graphene on SiO_2/Si at room temperature. Average carrier mobility is ~8800 $cm^2 V^{-1} s^{-1}$, indicated by black dash line. Inset: wafer-scale Hall bar arrays on graphene transferred by OVMs-modified supporting films (alpha-terpineol (10 wt%)/PMMA).

Supplementary Figure 12: Sheet resistance comparison of transferred graphene

The average sheet resistance of graphene on PET transferred by PPC/PMMA supporting films over a large area is 774 Ω/□, while the average sheet resistance of as-transferred graphene on PET by conventional PMMA-based transfer method is 1465 Ω/□ (Supplementary Fig. 12a-b). We believe the improved intactness and cleanness of graphene in our method ensure the reduced sheet resistance. Since the water and oxygen doping were strongly suppressed in our method, the graphene is intrinsic with a relatively low density of wrinkles and defects. Therefore, the additional doping by poly(3,4-ethylenedioxythiophene) (PEDOT): polystyrene sulfonate (PSS) was introduced to reduce the sheet resistance. In detail, by spin-coating the PEDOT:PSS layer on bare PET and as-transferred graphene on PET, the sheet resistance is 103 Ω/□ without graphene underneath (Supplementary Fig. 12c), and 87 Ω/□ with graphene underneath (Fig. 4e, main text), respectively.

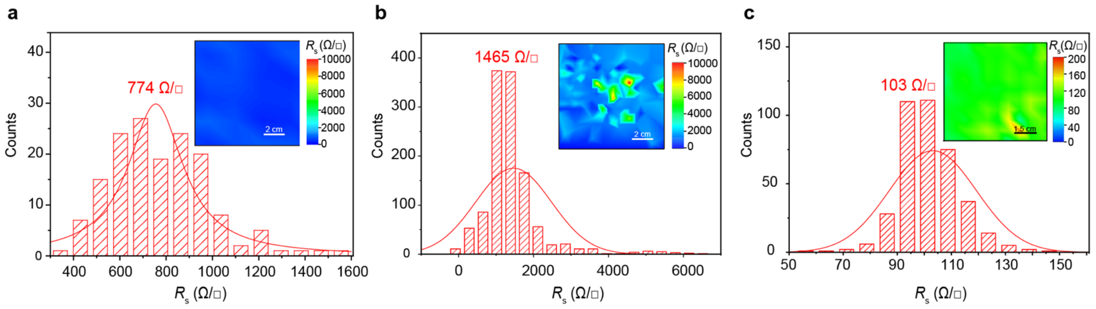

Supplementary Figure 12. Sheet resistance characterization. a-b. Statistics of graphene on PET substrates transferred by our method (**a**) and conventional PMMA-based transfer method (**b**). **c**. Sheet resistance statistics of the equivalent PEDOT:PSS layer on PET without graphene underneath. Inset: corresponding sheet resistance mapping.

Supplementary Figure 13: Raman characterization of as-fabricated MoS_2/graphene vertical heterostructure

We used the Raman spectroscopy to investigate the quality and interlayer coupling of as-fabricated MoS_2/graphene vertical heterostructure. As shown in Supplementary Fig. 13a, the uniform spatial distribution of E^1_{2g} intensity corresponding to the MoS_2 domains in inset of Fig. 5a in the main text confirms that no cracks and defect had been introduced into MoS_2 domains by the layer-by-layer transfer. Furthermore, no D band was observed over the imaging region, indicating that in transfer process of second layer MoS_2, no new cracks were formed in the underlying graphene (Supplementary Fig. 13b).

The enhanced interlayer interaction was confirmed by the observed photoinduced charge transfer. In our case, a uniform blueshift of the G band and redshift of the 2D band of graphene were observed (Supplementary Fig. 13c-e) This blueshift of the G band and redshift of the 2D band are caused by the additional doping in graphene through interlayer charge transfer, according to the reference[26-28]. Interlayer doping would also influence the FWHM of 2D band[19]. In addition, the compressive strain in graphene system[26], introduced by the conformal contact between MoS_2 and graphene, would also contribute to the observed broadening of the graphene 2D band (Supplementary Fig. 13f). In previously reported works, the MoS_2 was usually epitaxially grown on graphene surfaces with stronger interlayer coupling, which, however, usually introduces the defects in graphene, evidenced by enhanced D band intensity[29-31]. Meanwhile, when graphene surface is unclean with residues, the interlayer interaction would be hindered in the heterostructure fabricated by layer-by-layer transfer techniques[32]. Therefore, the observation clearly confirms the capability of our method in the van der Waals integration.

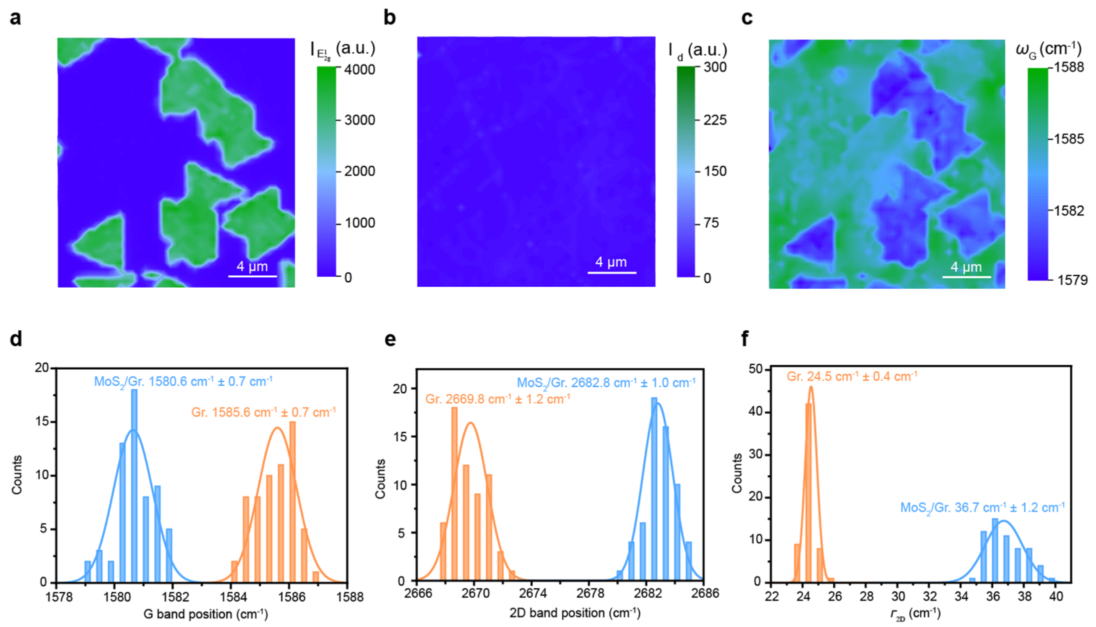

Supplementary Figure 13. Raman characterization of as-fabricated MoS_2/graphene vertical heterostructure. a-c, Corresponding Raman mapping of E^1_{2g} band intensity (**a**), D band intensity (**b**) and G band position (**c**). **d-f**, Statistical distribution of G band position (**d**), 2D band position (**e**), and the FWHM of 2D band (**f**) of graphene underneath the MoS_2 (blue) and bare graphene on SiO_2/Si (orange). MoS_2 and graphene were transferred by cedrol (10 wt%)/PMMA.

Supplementary Table 1: Time consumed in transfer of graphene onto PET substrates.

Transfer of A4-sized graphene films onto PET substrates

Step	Time (min)
I. Blade coating of supporting films	4
II. Lamination of TRT onto supporting films	1
III. Bubbling-based delamination of graphene	6
IV. Rinse of graphene films and drying	5
V. Lamination of graphene onto PET	1
VI. Releasing of TRT	1
VII. Heating and peeling of supporting films	3
Total	21

Supplementary Table 2: Time consumed in transfer of graphene onto SiO_2/Si substrates.

Transfer of 4-inch-sized graphene films onto SiO_2/Si substrates

Step	Time
I. Spin coating of supporting films	16 mins for four pieces
II. Lamination of TRT onto supporting films	4 mins for four pieces
III. Bubbling-based delamination of graphene	2 mins for four pieces
IV. Rinse of graphene films and drying	2 mins for four pieces
V. Lamination of graphene onto silicons	4 mins for four pieces
VI. Heating and peeling of supporting films	20 mins for four pieces
Total	48 mins for four pieces

Reference:

1. Li, Y. & Shimizu, H. Compatibilization by homopolymer: Significant improvements in the modulus and tensile strength of PPC/PMMA blends by the addition of a small amount of PVAc. *ACS Appl. Mater. Interfaces* **1**, 1650-1655 (2009).
2. Cui, S., Li, L. & Wang, Q. Enhancing glass transition temperature and mechanical properties of poly (propylene carbonate) by intermacromolecular complexation with poly (vinyl alcohol). *Compos. Sci. Technol.* **127**, 177-184 (2016).
3. Yoo, S. J., Lee, S. H., Jeon, M., Lee, H. S. & Kim, W. N. Effects of compatibilizers on the mechanical, morphological, and thermal properties of poly (propylene carbonate)/poly (methyl methacrylate) blends. *Macromol. Res.* **21**, 1182-1187 (2013).
4. Bae, S. *et al.* Roll-to-roll production of 30-inch graphene films for transparent electrodes. *Nat. Nanotechnol.* **5**, 574-578 (2010).
5. Haigh, S. J. *et al.* Cross-sectional imaging of individual layers and buried interfaces of graphene-based heterostructures and superlattices. *Nat. Mater.* **11**, 764-767 (2012).
6. Gao, L. *et al.* Repeated growth and bubbling transfer of graphene with millimetre-size single-crystal grains using platinum. *Nat. Commun.* **3**, 1-7 (2012).
7. Lin, L. *et al.* Towards super-clean graphene. *Nat. Commun.* **10**, 1-7 (2019).
8. Zhang, Z. *et al.* Rosin-enabled ultraclean and damage-free transfer of graphene for large-area flexible organic light-emitting diodes. *Nat. Commun.* **8**, 1-9 (2017).
9. Cunge, G. *et al.* Dry efficient cleaning of poly-methyl-methacrylate residues from graphene with high-density H_2 and H_2-N_2 plasmas. *J. Appl. Phys.* **118**, 123302 (2015).
10. Zhu, W. *et al.* Structure and electronic transport in graphene wrinkles. *Nano Lett.* **12**, 3431-3436 (2012).
11. Deng, B. *et al.* Wrinkle-free single-crystal graphene wafer grown on strain-engineered substrates. *ACS Nano* **11**, 12337-12345 (2017).
12. Gammelgaard, L. *et al.* Graphene transport properties upon exposure to PMMA processing and heat treatments. *2D Mater.* **1**, 035005 (2014).
13. Demirbaş, T. & Baykara, M. Z. Nanoscale tribology of graphene grown by chemical vapor deposition and transferred onto silicon oxide substrates. *J. Mater. Res.* **31**, 1914-1923 (2016).

14. Kim, H. *et al.* Copper-vapor-assisted chemical vapor deposition for high-quality and metal-free single-layer graphene on amorphous SiO_2 substrate. *ACS Nano* **7**, 6575-6582 (2013).
15. Yuan, G. *et al.* Proton-assisted growth of ultra-flat graphene films. *Nature* **577**, 204-208 (2020).
16. Gao, L. *et al.* Face-to-face transfer of wafer-scale graphene films. *Nature* **505**, 190-194 (2014).
17. Wang, M. *et al.* Single-crystal, large-area, fold-free monolayer graphene. *Nature* **596**, 519-524 (2021).
18. Neumann, C. *et al.* Raman spectroscopy as probe of nanometre-scale strain variations in graphene. *Nat. Commun.* **6**, 1-7 (2015).
19. Lee, J. E., Ahn, G., Shim, J., Lee, Y. S. & Ryu, S. Optical separation of mechanical strain from charge doping in graphene. *Nat. Commun.* **3**, 1-8 (2012).
20. Ferrari, A. C. *et al.* Raman spectrum of graphene and graphene layers. *Phys. Rev. Lett.* **97**, 187401 (2006).
21. Kim, S. J. *et al.* Ultraclean patterned transfer of single-layer graphene by recyclable pressure sensitive adhesive films. *Nano Lett.* **15**, 3236-3240 (2015).
22. Hong, J. Y. *et al.* A rational strategy for graphene transfer on substrates with rough features. *Adv. Mater.* **28**, 2382-2392 (2016).
23. Zhao, H. *et al.* PMMA direct exfoliation for rapid and organic free transfer of centimeter-scale CVD graphene. *2D Mater.* **9**, 015036 (2021).
24. Seo, Y.-M. *et al.* Defect-free mechanical graphene transfer using n-doping adhesive gel buffer. *ACS Nano* **15**, 11276-11284 (2021).
25. Zhang, X. *et al.* A scalable polymer-free method for transferring graphene onto arbitrary surfaces. *Carbon* **161**, 479-485 (2020).
26. Rao, R. *et al.* Spectroscopic evaluation of charge-transfer doping and strain in graphene/MoS_2 heterostructures. *Phys. Rev. B* **99**, 195401 (2019).
27. Ago, H. *et al.* Controlled van der Waals epitaxy of monolayer MoS_2 triangular domains on graphene. *ACS Appl. Mater. Interfaces* **7**, 5265-5273 (2015).

28. Zhou, K.-G. *et al.* Raman modes of MoS_2 used as fingerprint of van der Waals interactions in 2-D crystal-based heterostructures. *ACS Nano* **8**, 9914-9924 (2014).

29. Hoang, A. T. *et al.* Epitaxial Growth of Wafer-Scale Molybdenum Disulfide/Graphene Heterostructures by Metal–Organic Vapor-Phase Epitaxy and Their Application in Photodetectors. *ACS Appl. Mater. Interfaces* **12**, 44335-44344 (2020).

30. Shi, Y. *et al.* van der Waals epitaxy of MoS_2 layers using graphene as growth templates. *Nano Lett.* **12**, 2784-2791 (2012).

31. McCreary, K. M. *et al.* Large-Area Synthesis of Continuous and Uniform MoS_2 Monolayer Films on Graphene. *Adv. Funct. Mater.* **24**, 6449-6454 (2014).

32. Alexeev, E. M. *et al.* Imaging of interlayer coupling in van der Waals heterostructures using a bright-field optical microscope. *Nano Lett.* **17**, 5342-5349 (2017).
